# Molecular determinants underlying functional innovations of TBP and their impact on transcription initiation

**DOI:** 10.1038/s41467-020-16182-z

**Published:** 2020-05-13

**Authors:** Charles N. J. Ravarani, Tilman Flock, Sreenivas Chavali, Madhanagopal Anandapadamanaban, M. Madan Babu, Santhanam Balaji

**Affiliations:** 1 MRC Laboratory of Molecular Biology, Francis Crick Ave, Cambridge, UK CB2 0QH; 2 Fitzwilliam College of the University of Cambridge, Storey’s Way, Cambridge, CB3 0DG UK; 3 Department of Biology, Indian Institute of Science Education and Research (IISER) Tirupati, Tirupati, Andhra Pradesh India

**Keywords:** Protein sequence analyses, Molecular evolution

## Abstract

TATA-box binding protein (TBP) is required for every single transcription event in archaea and eukaryotes. It binds DNA and harbors two repeats with an internal structural symmetry that show sequence asymmetry. At various times in evolution, TBP has acquired multiple interaction partners and different organisms have evolved TBP paralogs with additional protein regions. Together, these observations raise questions of what molecular determinants (i.e. key residues) led to the ability of TBP to acquire new interactions, resulting in an increasingly complex transcriptional system in eukaryotes. We present a comprehensive study of the evolutionary history of TBP and its interaction partners across all domains of life, including viruses. Our analysis reveals the molecular determinants and suggests a unified and multi-stage evolutionary model for the functional innovations of TBP. These findings highlight how concerted chemical changes on a conserved structural scaffold allow for the emergence of complexity in a fundamental biological process.

## Abstract (web summary)

The TATA-box binding protein (TBP) is required for transcription initiation in archaea and eukaryotes. Here the authors delineate how TBP’s function has evolved new functional features through context-dependent interactions with various protein partners.

## Introduction

**Fig. 1:**
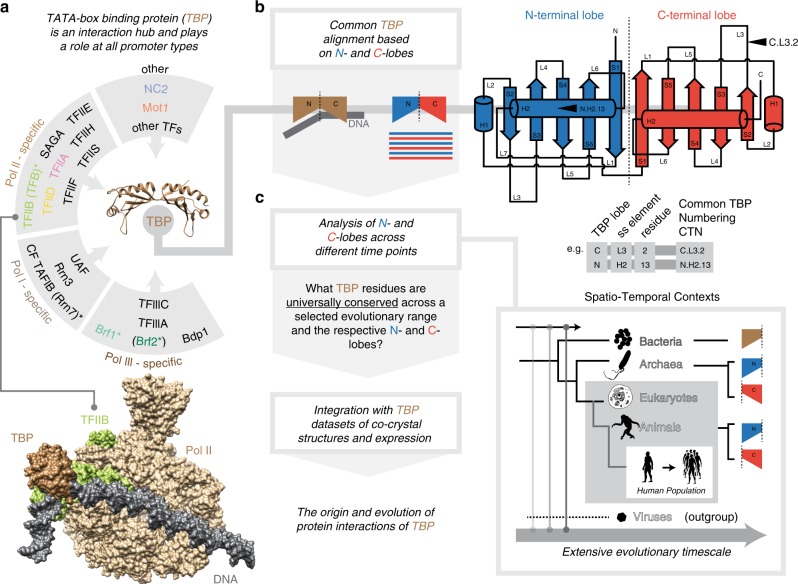
Schematic overview of interactions of TBP, RefMSA, and the spatio-temporal analyses. **a** The interaction overview of TBP (shown in dark brown) depicting the structure of TBP and its interactions with various components of the transcription initiation complexes in eukaryotes. The bottom inset highlights the structural view of TBP associations with DNA (gray) and RNA polymerase II (beige) mediated by TFIIB (green). **b** A comprehensive common TBP reference multiple sequence alignment (RefMSA) at the level of TBP-lobes (N-terminal and C-terminal lobes indicated in “blue” and “red”, respectively) was constructed based on an initial structural alignment of TBP-lobes (see “Methods” section, Supplementary Fig. 2a and Supplementary Data 16 for details). The initial structural alignment of TBP lobes was used to develop a common TBP numbering (CTN) scheme for any residue in the RefMSA. This is illustrated in the example just below the cartoon as three elements for every residue position. The first element of a CTN defines the lobes (N or C), the second refers to the secondary structure element (e.g. L1 for loop 1, H2 for helix 2, etc.) and the third position represents the position in the specified secondary structure element. Thus in the given example an alignment position N.H2.13 would refer to the 13th position in helix H2 of the N-terminal lobe (see Supplementary Fig. 2a). **c** The approach employed in the study involves traversing multiple phylogenetic ranges (temporal), as well as considering different sets of TBP-lobe sequences (spatial) to uncover conserved molecular signatures.

Transcription initiation in eukaryotes and archaea relies on a central molecule called TATA-box-binding protein (TBP) that recruits additional factors (e.g. general transcription factors and RNA polymerase) to assemble the pre-initiation complex (PIC)^1–7^. TBP is a relatively small protein that is shaped like a saddle and contains two lobes (TBP lobes) that bind to specific DNA sequences in the gene promoter. Both of the TBP lobes (N-terminal and C-terminal lobes) belong to the helix-grip fold and are “joined together” to form a single protein^8–11^ (Fig. 1a, b). TBP is the essential component even in the simplest form of the PIC as seen in archaea^12^. Upon DNA binding, TBP recruits an adapter protein called TFB that in turn interacts with the RNA polymerase to initiate transcription^1,13–15^. In eukaryotes, the process of transcription has diversified with three different RNA polymerases (RNA Pol I, II, and III) that transcribe distinct types of genes, such as tRNAs, mRNAs, and rRNAs^4,16–20^. Despite this diversification, TBP has remained a central component for assembling different sets of proteins that eventually recruit the different polymerases (Supplementary Fig. 1)^17,21,22^.

How does the same TBP molecule recruit different RNA polymerases in eukaryotes? Biochemical and structural studies have revealed a number of factors that serve as adapters to interact with TBP and the three different polymerases. For instance, compared to a single TFB in archaea, eukaryotes contain multiple paralogs, such as Rrn7p, TFIIB, and Brf1/2 that interact with Pol I, II, and III respectively^23–27^. Furthermore, regulatory proteins such as BTAF1/Mot1p and NC2 (DR1-DRAP) can interact with TBP and evict them from the promoter^28–32^. This allows for extensive regulation of gene expression at different promoters. Thus during evolution, the key interaction between TBP and TFB as seen in archaea appears to have diversified into a complex network of interactions involving multiple paralogous proteins and new regulators to maintain fidelity in recruiting the different types of polymerases but still use the same TBP^3,21,22,28,29,33,34^ (Supplementary Fig. 1). This raises the question how such a central molecule evolved to adapt to this increasing complexity of interactions from archaea to eukaryotes?

A recent study by Kawakami et al. ^35^ that analyzed archaeal and eukaryotic TBP and TFIIB proteins proposed that asymmetric evolution has contributed to the complexity of the eukaryotic transcriptional system. Specifically, the study suggested that TBP evolved in two stages: one where eukaryotic TBP initially acquired multiple eukaryote-specific interactors through asymmetric evolution of the two repeats (or lobes), and the other where its diversification halted and its asymmetric structure spread throughout eukaryotic species. However, the different interactors of TBP emerged at various different points in evolution^3^, and many of them of are encoded by unrelated sequences, raising the question how they were progressively integrated into the transcriptional system. Hence, there is a requirement for unified analyses by taking into consideration the evolutionary divergence of TBP-interacting factors. Beyond the asymmetric sequence divergence, several organisms have evolved multiple paralogs of TBP, and they have all acquired multiple protein regions in the N-terminus, raising the question of their contribution to the complexity of eukaryotic transcriptional systems. These considerations suggest that TBP evolution is likely to involve multiple steps at different time points, characterized by the emergence of specialized functional innovations that is driven by TBP-interacting factors, all contributing to the currently observed complexities of eukaryotic transcription initiation.

In this study, we identified and compared TBP, TBP paralogs, and TBP-like proteins from eukaryotes, archaea, bacteria, and viruses. Based on this, we inferred sequence segments and key positions on TBP in the different regions that are critical for its diverse functions, such as interaction with DNA, adapter molecules, and regulators, to explain the distinct functional roles of TBP. Because the functions emerged at different points in time during evolution and their interaction sites are distributed on the TBP molecule, we devised an approach to infer specific sets of residues that emerged at these time points and linked them to the specific function (i.e. molecular signature positions; Fig. 1c) by comparing against sequences that emerged early in evolution (e.g. bacteria) and by using viruses as outgroups. We integrated various datasets such as the TBP co-complex structures with: different interaction partners, large-scale protein interaction networks, protein expression data, published biochemical mutational data, cancer mutations and natural variation of TBP and associated factors in human populations. We also validated and present the molecular determinants of the functional innovations and comprehensively define a unified and multi-stage evolutionary trajectory of TBP.

## Results

### A universal TBP-lobe-level alignment enables residue-level interpretation of function

To enable comparative analyses of protein sequences between organisms across a diverse phylogenetic range, it is important to construct a unified alignment of TBP-like protein sequences from different lineages. For this, we first built a comprehensive structural alignment of the entire pseudo-symmetric TBP molecule based on known structures of TBP. Using this alignment, we could identify the N-terminal and C-terminal lobe boundaries, which we then treated as separate units to prepare the initial TBP-lobe level alignment (Supplementary Fig. 2a, b and https://www.mrc-lmb.cam.ac.uk/genomes/tbp/). Using the above alignment as a basis, we identified orthologs and paralogs of human TBP from completely sequenced genomes representing major lineages of life: bacteria, archaea, and eukaryotes (Fig. 1b,c). The identified orthologs and paralogs were integrated into the alignment at the level of individual TBP lobes (see “Methods” section).

The TBP-lobe level alignment allowed us to build a comprehensive sequence profile that enabled sensitive sequence-based profile searches, resulting in the identification of more distantly related sequences that contain a TBP-lobe in bacteria and TBP-like sequences in diverse viruses. The sequence searches allowed us to build a comprehensive multiple sequence alignment for TBP-lobe sequences covering diverse lineages and representing broad evolutionary diversity within each lineage. This resource, which we make available to the community (https://www.mrc-lmb.cam.ac.uk/genomes/tbp; Supplementary Data 1), is referred to as RefMSA and contains a total of 218 TBP-lobe sequences (119 protein sequences, 105 organisms: 24 eukaryotes, 24 archaea, 19 bacteria, and 38 viruses). We also provide an all-inclusive TBP-lobe level sequence alignment that contains more than 400-lobe sequences from over 170 organisms at the same web resource. These alignments contain 209 alignment positions. Importantly, the RefMSA enabled us to define the structurally equivalent residues in the TBP-lobes among sequences that have diverged quite extensively and were not identifiable through standard sequence searches (Supplementary Fig. 2b). The RefMSA also enabled the consistent mapping of point mutations from existing functional studies in TBP from diverse organisms.

To enable the comparison of any residue/position between the different TBP-lobe sequences in the RefMSA, we developed a common TBP-lobe numbering (CTN) system, by integrating consensus secondary structure information of available crystal structures of the TBP lobes from the initial structural alignment with the RefMSA (Supplementary Fig. 2a). Briefly, the CTN is composed of three fields that help to locate the position of a residue on a TBP-lobe. The first field refers to the N-terminal or the C-terminal lobe (which is not applicable to sequences with a single TBP-lobe). The second field refers to the secondary structure element (strands 1–5, S1–S5; helices 1–2, H1–H2; loops 1–6, L1–L6) and the third field refers to the position within the secondary structure element. For instance, C.L3.2 refers to the second position in loop L3 of the C-terminal lobe of TBP (Supplementary Fig. 2a).

The comprehensive RefMSA resource allowed us to extract selected sets of TBP-lobe sequences (e.g. of just the N-terminal or C-terminal TBP-lobe; spatial context), as well as restrict the analyses to distinct phylogenetic ranges (e.g. only archaea and eukaryotes; temporal context). By defining different spatio-temporal contexts, we could identify universally conserved positions within this spatio-temporal context. The residues in these positions therefore constitute a set of signatures. A subset of these signature positions might be critical for maintaining the fold whereas the other signature positions constitute residues that can be linked to specific functions (i.e. the ability to interact with different biomolecules, such as DNA, general transcription factors, eviction factors, etc.) that emerged during TBP evolution (Supplementary Fig. 2c).

### Signatures of nucleic acid recognition by TBP-lobe sequences

**Fig. 2:**
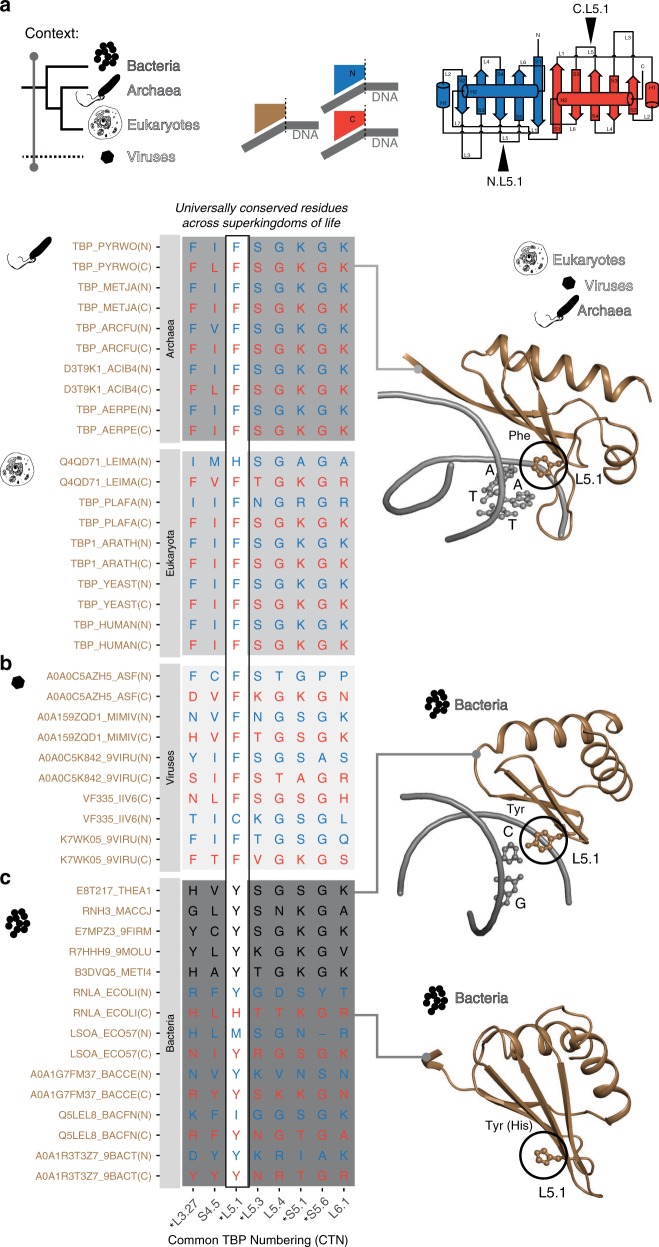
Universally conserved residues of TBP-lobe like regions and their functional relevance. **a** The spatio-temporal context denotes a phylogenetic range of all the three superkingdoms of life (temporal) and the consideration of entire TBP lobe-like regions (spatial). NA-binding residues are highlighted with ‘*’. CTN position L5.1 contains an almost universally conserved Phe residue in eukaryotes and archaea. Residues at L5.1 are shown in ball and stick representation, and highlighted with black circles on the structures (right). **b** Universally conserved positions are moderately preserved in TBP lobe-like sequences of dsDNA viruses and the most prominent L5.1 position is a Phe in most viruses. This suggests TATA-box sequence binding potential for viral TBP-like sequences. **c** In bacteria, L5.1 position predominantly contains a conserved Tyr or a His as opposed to Phe at this position in archaea, eukaryotes, and viruses. The structures of TBP lobe-like regions show that the aromatic residues of L5.1 are oriented in a similar manner across the three superkingdoms. The PDB identifiers for structures are 1cdw, 4py5, and 4i8o from top to bottom.

To identify the signatures of nucleic acid-binding function of TBP, we first defined a spatio-temporal context by inspecting a subset of the RefMSA that included all TBP-lobe sequences from archaea, eukaryotes, and bacteria. Out of the 209 positions in the RefMSA TBP-lobe alignment, we identified eight residue positions that are universally conserved (Fig. 2 and Supplementary Fig. 2a).

### Eukaryotes and archaea

Analysis of the known structures of TBP in complex with the DNA revealed that the majority of them are located in the nucleic acid-binding interface (five residue positions; *p*-value = 0.009 hyper-geometric test; Fig. 2a, Supplementary Data 1; the other three positions may be involved in maintaining the protein fold). Of particular interest is position L5.1, which contains a highly conserved aromatic residue (Phe) in archaea and eukaryotes (both in the N-terminal and C-terminal lobes). Given that Phe is found at L5.1 in over 90% of sequences in the alignment, it is likely that this residue is a crucial contributor for binding DNA, specifically sequences containing the TATA-box motif^16,36^. Consistent with this, experiments where this Phe was altered to another aromatic residue, e.g. Tyr, have shown to influence the binding of TBP to TATA-containing sequences^16,36^.

### Viruses

The discovery of diverse TBP-like sequences (i.e. a single sequence with both N-terminal and C-terminal lobes as in TBP) in viruses as reported in the RefMSA allowed us to investigate the importance of these universally conserved residues in nucleic acid binding. While the presence of TBP-like sequences in viruses has been documented (for example, C7U0H2_9PHYC, a viral sequence in the RefMSA, has been annotated to contain TBP like domain in the UniProt database), the RefMSA contains 84 viral sequences (at the lobe level) from 38 different viruses, including the highly divergent versions of TBP in poxviruses that were not annotated before (see Supplementary Data 1 and 2). Interestingly, all the viruses that contained a TBP-like sequence belonged to the class of double-stranded DNA-binding viruses. The above-mentioned five DNA-binding positions show conservation from 50% to 90% suggesting a moderate level of conservation, whilst strikingly, position L5.1 (Phe) is conserved in 90% of the viral sequences (see Fig. 2b). This implies that viral TBP-like sequences may regulate the expression of their own genes and possibly, as well as that of the host. Thus, despite the evolutionary pressure for viruses to evolve their sequences rapidly, the five positions that contact the DNA have been highly conserved. This highlights the fundamental role of these positions in recognizing DNA sequence.

### Bacteria

Unlike viruses, the bacterial homologs do not contain TBP-like sequences (two-lobe containing sequences). The initial sequence search retrieved bacterial sequences for which structures of the TBP-lobe like region exist in the PDB, including one in complex with an RNA–DNA duplex. This allowed us to map signature residues that enable the TBP-lobe to interact with nucleic acids in cellular organisms (Fig. 2c). An analysis of the annotation of the bacterial sequences in the RefMSA revealed that the sequences belong to different families: the RNAse H3 family^37^, which surprisingly spans a significant phylogenetic range in bacteria; e.g. Desulfurobacterium, Firmicutes, Chlamydia, Tenericutes, Bacteroidetes, and Verrucomicrobia (Fig. 2c, Supplementary Data 1) and the rnlA toxin family (i.e. in the toxin/anti-toxin system; Fig. 2c). Although the bacterial homologs do not have the characteristic pseudo-symmetric architecture of the classical TBP found in archaea and eukaryotes, they could still mediate interaction with double-stranded nucleic acids as in the case of RNAse H3 (which binds a DNA–RNA duplex to cleave RNA), and the rnlA toxins (endoribonucleases that act on mRNA in bacterial cells thereby acting against the invading bacteriophages^38^). An integrated analysis of the sequences of the distantly related members revealed that there is also a highly conserved aromatic residue, notably a conserved Tyr at L5.1 (Fig. 2c). From a structural point of view, the L5.1 residue similarly protrudes from the loop (L5), consistent with the configuration in eukaryotes and archaea in both the rnlA toxin as well as in the RNAse H3 structure. In the RNAse H3 structure, this position directly contacts the RNA–DNA duplex in the minor groove, just like how TBP binds the DNA duplex.

Taken together, the universally conserved positions across a wide set of cellular organisms (and viruses) in the RefMSA of TBP-lobe appear to constitute an ancient double-stranded nucleic acid-binding module and was also used for nucleic acid binding in bacteria albeit in a different context. Thus, duplication and fusion of a TBP-lobe sequence that functioned as a double-stranded nucleic acid-binding module might have resulted in the emergence of classical TBP-like sequences in archaea and eukaryotes^37^.

### Signatures in the C-terminal lobe of TBP-like sequences for interacting with adapters

**Fig. 3:**
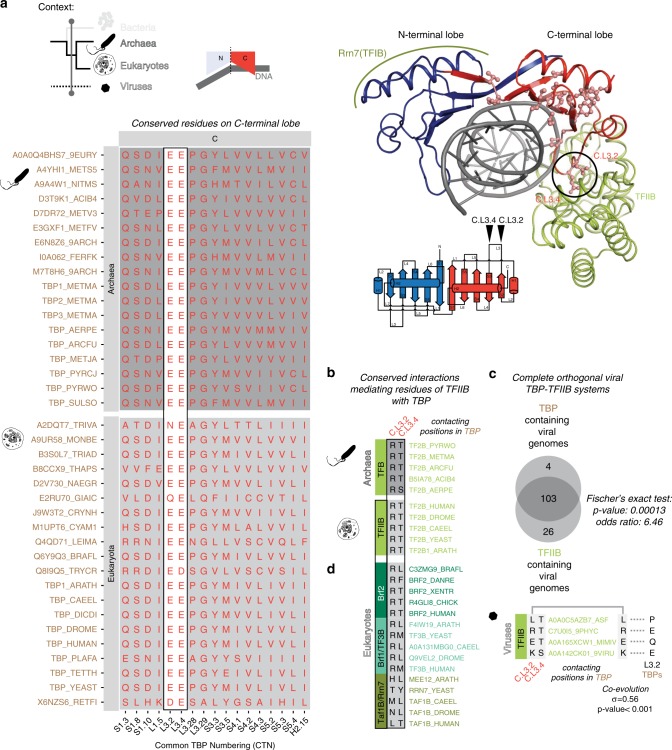
C-terminal lobe-specific signatures of TBP and their interaction with TFIIB homologs. **a** The spatio-temporal context is confined to archaea and eukaryotes (temporal) and the C-terminal TBP-lobes (spatial). The conserved residues are mapped on to TBP–TFIIB complex structure (bottom right; PDB identifier: 1c9b). The most prominent conserved glutamate residues at C.L3.2 and C.L3.4 that mediate interactions with TFIIB are highlighted in a box. **b** TFB in archaea and its eukaryotic ortholog TFIIB have conserved Arg and Thr that interact, respectively, with conserved glutamate residues at C.L3.2 and C.L3.4 of TBP C-terminal lobes. **c** Orthogonal TBP–TFIIB system in dsDNA viruses. The Venn diagram depicts the shared presence of TBP and TFIIB homologs in viral genomes. A large number of viral genomes contain both TBP and TFIIB homologs more often than expected by chance (Fisher’s exact test). The residues at C.L.3.2 of viral TBPs C-terminal lobe and equivalent residues in the viral TFIIB orthologs of the Arg residue in eukaryotic TFIIBs that interact with C.L.3.2 TBP residue are shown to co-evolve (see “Methods” section). **d** Eukaryotic paralogs of TFIIB, i.e. Brf1/2 (Pol III system) contain a conserved Arg that could interact with Glu (C.L3.2) of TBP, which is also observed in co-complex structures (PDB identifier: 6f40 and 4roc, respectively). Human TAF1B or yeast Rrn7p (Pol I system) and its homologs do not possess the critical Arg that could interact with Glu of C.L3.2.

To detect the C-terminal lobe-specific signatures that contribute to its ability to interact with adapters, such as TFIIB, we considered a spatio-temporal context consisting of archaeal and eukaryotic C-terminal TBP-lobe sequences only and excluded the N-terminal TBP-lobe sequences and bacterial TBP-lobe sequences. We identified 17 positions that showed high conservation in the C-terminal lobe but were not conserved (or were conserved as a different residue) in the N-terminal lobe (Fig. 3a). To assess their functional relevance, we then mapped these positions on available crystal structures of TBP–TFIIB complexes and their orthologous archaeal complex TBP–TFB (the TFIIB equivalent adapter protein). Interestingly, 8 of the 17 residue positions are involved in maintaining the fold of the C-terminal lobe, while six positions are involved in binding DNA. However, two signature acidic residues (C.L3.2 and C.L3.4; both primarily glutamate residues) were directly involved in the TFIIB/TFB interactions (Fig. 3a). These two residues (C.L3.2 and C.L3.4) interact with Arg and Thr, respectively, in TFIIB/TFB. A comprehensive sequence alignment of TFIIB/TFB homologs (Supplementary Data 3) revealed that the Arg and Thr are highly conserved. Consistent with their role, these glutamate-containing positions were not conserved in the bacterial TBP-lobe sequences, which do not have direct TFIIB homologs (although sigma factors are distant homologs of TFIIB, Supplementary Data 4). This suggests that the interactions between the charged residues in C.L3.2 and C.L3.4 with Arg and Thr in the TFIIB/TFB orthologs evolved in the last common ancestor of archaea and eukaryotes, and are highly conserved across all the major lineages in eukaryotes and archaea (Fig. 3b). Hence, it appears that TBP acquired the function of interacting with the TFIIB/TFB adapter, cementing its role in the central process of transcription initiation.

By analyzing the viral TBP-like sequences from the RefMSA, we observed that positions C.L3.2 and C.L3.4 show greater variability than their homologs in archaea and eukaryotes (Supplementary Fig. 3). Some viral sequences conserve the nature of the negatively charged amino acid at the above signature positions, whereas the others have completely different residues at these positions. These observations imply that the viral TBPs are unlikely to interact with eukaryotic TFIIBs or their homologs (conservation at C.L3.2 is ~40%; Supplementary Data 1 and 2). This prompted us to investigate the presence of TFIIB-like proteins in the viral genomes. Strikingly, for almost all viruses that had a TBP-like sequence, we could detect a TFIIB-like protein in their genomes (Supplementary Data 5). This implies that a large portion of viral TBPs are likely to interact with their own, viral genome encoded TFIIB-like proteins. By building an alignment of TFIIB-like sequences in addition to the TBP-like sequences from the different viral genomes, we analyzed patterns of co-evolution of the signature interacting positions (Supplementary Data 6). We observed that C.L3.2 and C.L3.4 in viral TBP-like sequences co-evolve with the Arg or Thr equivalent positions in viral TFIIB-like sequences (correlation coefficient of 0.56; *P*-value < 0.001; Fig. 3c). This suggests that most viral genomes investigated here are likely to have a complete, orthogonal TBP–TFIIB-like system, possibly interacting in a similar manner but unlikely to be influenced by the host TBP–TFIIB system.

In contrast to archaea, eukaryotic TBP interacts with TFIIB paralogs, such as Brf1 and Brf2 to recruit RNA polymerase III and TAF1B/Rrn7p to recruit RNA polymerase I. An analysis of the orthologous sequences of Brf1 and Brf2 revealed that the position equivalent to the Arg in TFIIB, which is known to interact with TBP position C.L3.2, is also highly conserved. The position equivalent to the Thr residue that interacts with TBP C.L3.4 is also highly conserved in Brf2 but is more divergent in Brf1 (Fig. 3d). Using the structure of TBP–TFIIB, we identified all interface positions involved in this interaction. A more detailed analysis of all the interface positions among their respective eukaryotic orthologs revealed that the interacting residues on TBP are much more conserved among orthologs than the interacting residues of TFIIB (as measured by BLOSUM score; Supplementary Fig. 4a; Supplementary Data 4). Interestingly, overall, the TBP-interacting positions of TFIIB when mapped on to Brf1 and Brf2 are much more variable in terms of conservation among their respective orthologs than in TFIIB (Supplementary Fig. 4a). This indicates that the TBP interacting residues on the TFIIB paralogs, Brf1 and Brf2, have diverged but still recognize the same conserved interface on TBP. Consistent with this, an analysis of human natural variation data from over 100,000 individuals showed that the TBP-interacting positions on TFIIB paralogs show an increased number of missense mutations in the human population compared to the interface positions on TBP (Supplementary Fig. 4b).

These observations collectively suggest that using a conserved set of surface TBP residues, which include the signature positions in the C-terminal lobe, the same TBP molecule can interact with a distinct set of adapters, such as TFIIB and Brf1/Brf2, and recruit different molecular machines to initiate transcription from a distinct set of promoters. In other words, TFIIB and their paralogs act as a bridge through distinct characteristic interfaces to link TBP to different RNA polymerase systems. Intriguingly, when TBP-interacting positions of TFIIB were mapped onto TAF1B, the TFIIB paralog that recruits RNA polymerase I, a distinct pattern of conservation, which does not involve the conserved Arg or Thr that mediate interactions with TBP C.L3.2 or C.L.3.4, respectively, was observed. Therefore TAF1B or its orthologs are unlikely to interact with TBP in the same way. In support of this possibility, chemical cross-linking data^39^ show that the yeast ortholog (Rrn7p) of human TAF1B interacts with N-terminal lobe of yeast TBP but not with C-terminal lobe of yeast TBP. However, there might be alternative mechanisms that facilitate transcription initiation events at Pol I promoters that do not involve TBP^40^ and this may be reflected in the observation that the Rrn7p/TAF1B–TBP-binding interface is the most rapidly evolving among the different adapters (Supplementary Fig. 4a). These observations are in general agreement with earlier studies that have suggested that the components of Pol I system are more divergent and rapidly evolving as compared to their counterparts in the Pol II and Pol III systems^41,42^

Taken together, the presence of the highly conserved acidic signature residues C.L3.2 and C.L3.4 across different organisms suggests that these C-terminal lobe signature positions emerged to enable a key function of TBP, which is to mediate interactions with diverse adapter molecules, such as TFIIB and Brf1/Brf2 to initiate transcription. Thus, TBP uniquely interacts with each of the different polymerase systems through distinct adapters (TFIIB homologs) and this requirement might have constrained the TBP sequence to be highly conserved at these sites.

### Signatures of the eukaryotic N-terminal lobe for interacting with gene expression regulators

**Fig. 4:**
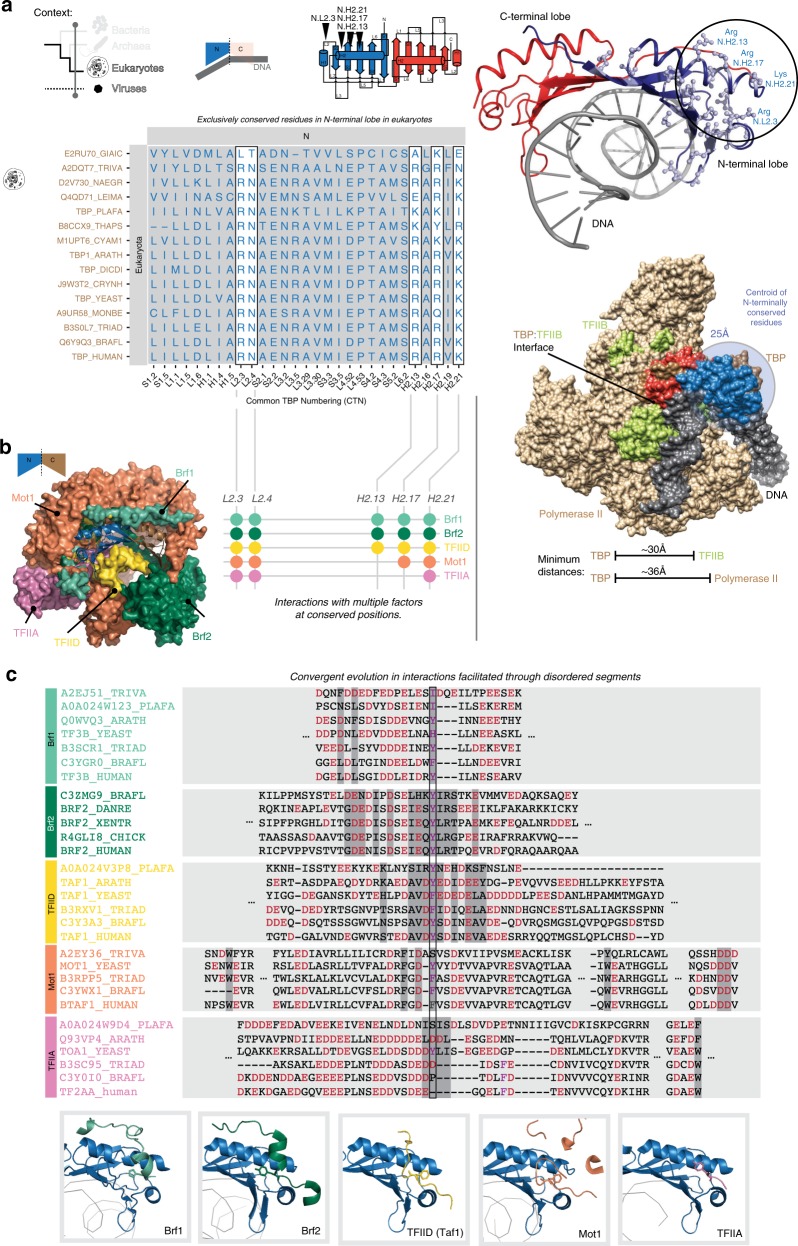
N-terminal lobe-specific signatures of TBP and convergent evolution among TBP-interacting proteins. **a** The spatio-temporal context includes only eukaryotes (temporal) and the N-terminal TBP-lobes (spatial). Exclusively conserved residues in the N-terminal lobe of TBP in eukaryotes are shown. This includes five highly conserved positively charged or Asn-containing positions (marked with boxes). These five residues cluster in 3-D space as indicated by mapping on to a TBP structure (top right; PDB identifier: 1cdw). **b** A surface representation of TBP in complex with factors that interact with the N-terminal lobe of TBP (shown in different colors). This indicates the existence of common and overlapping interaction sites on the N-terminal lobe of TBP for the various interacting factors (left). Filled circles (right) represent the molecular signature positions of TBP that are involved in interactions with residue(s) of the various factors. **c** Interacting regions of the various factors that interact with the N-terminal lobe of TBP are displayed as sequence alignments (top). These regions are within a 10-residue window around residues that directly contact TBP the co-complex structures (shaded in dark gray). The chemical nature of these residues, acidic (red) and aromatic (purple), is conserved despite low conservation at the level of residue identity and being intrinsically disordered. The insets below depict the “snapshot view” of the interaction between the factors (bottom; in different colors) interacting with the N-terminal lobe region (shown in blue) of TBP (bottom). The PDB identifiers for the co-complex structures in the insets are 1ngm (Brf1), 4roc (Brf2), 4b0a (TAF1–TFIID), 3oc3 (Mot1p), and 1nh2 (TFIIA).

In eukaryotes, the N-terminal lobe of TBP is known to interact with several general transcription factors, such as TFIIA, TFIID (TAF1 subunit), Brf1 and Brf2 as well as the TBP evicting factor BTAF1/Mot1p and NC2^21,28,29,34,43–46^. To detect signatures of the N-terminal lobe, we defined a spatio-temporal context by only analyzing eukaryotic N-terminal lobe sequences of TBP from the RefMSA. This allowed us to identify 29 highly conserved positions that are unique to the N-terminal lobe, i.e. not conserved or conserved as a different residue in the C-terminal lobe (Fig. 4a). While 13 positions are important for the fold and three positions for DNA binding, we found five positions that spatially cluster on the N-terminal lobe 4 of which are positively charged residues (N.L2.3, N.H2.13, N.H2.17, and N.H2.21). The positively charged cluster is solvent exposed and located farthest away from Pol II holoenzyme-binding regions (~36 Å away) and the site of the TFIIB interaction (~30 Å away). Such an arrangement offers a conserved binding interface for other factors while not interfering with the recruitment of the polymerase via TFIIB (Fig. 4b).

To understand the functional relevance of these positions, we mapped them to the available structures of TBP in complex with diverse factors such as TFIIA, BTAF1/Mot1p, Brf1, Brf2, and TAF1. The five positions of the N-terminal lobe signature were directly involved in interactions with these factors (Fig. 4b, Supplementary Fig. 5). Thus, the different factors that can have antagonistic outcomes on transcription initiation are likely to compete for the same interface to interact with TBP (Supplementary Data 7 and 8). Although these interacting factors belong to different protein folds, we found marked similarities between them in terms of their general sequence composition at the interaction interface. Specifically, the interacting residues of the factors were aromatic or acidic and “contacted” positively charged residues, especially the N-terminal lobe signature residues that form the highly conserved positively charged cluster on TBP (Fig. 4b, Supplementary Fig. 5).

We constructed the multiple sequence alignment of TFIIA, BTAF1/Mot1p, Brf1, Brf2, and TAF1 across the major eukaryotic lineages (Supplementary Data 7) and analyzed the residues in the factors around the interface positions. We found that they were enriched in negatively charged residues with a key aromatic residue that forms the center of interaction with TBP. Thus, it appears that the uniting theme is in the interaction interface between TBP and these factors are characterized by local charge of the interacting peptide on the factors rather than the precise sequence conservation (Fig. 4c). While electron cryo-microscopy and crystal structures present a more static view of the complexes, given the nature of the residues at the interface, it appears that conservation of chemistry around these positions may contribute to a more dynamic interaction. Such polypeptide sequences are likely to be disordered in their native form and become structured upon interacting with TBP. Indeed we found that interacting residues were in stretches that are predicted to be disordered (probability value (*p*) > 0.5) (Supplementary Fig. 6). Consistent with this observation, we found that the interface position of TBP is more conserved than the TBP-interacting interface positions on the factors in eukaryotes. (Supplementary Fig. 7a). This trend was also seen when we investigated natural human genetic variation data where the interface positions of the factors had more genetic variation than the interface positions of TBP (Supplementary Fig. 7b). Collectively, these findings suggest a recurring theme of interacting residues in the factors is fast evolving compared to that of TBP.

A detailed analysis of the N-terminal lobe of viral TBP-like sequences revealed that the signature residues of the eukaryotic N-terminal lobe are not conserved (Supplementary Fig. 8). More specifically, this implies that viral TBP is unlikely to interact with the above-mentioned host regulatory factors. Thus, viral TBP-like sequences might evade host regulatory proteins that can inhibit transcription initiation, especially that of BTAF1/Mot1p’s ability to evict TBP.

Taken together the overlapping site of interaction between several factors with the N-terminal TBP-lobe signature of positively charged patches indicates that this TBP signature could act as a critical regulatory interface. Such an interface can modulate gene expression by facilitating competing or co-operative interactions, and can be exploited in nature for influencing stochasticity in gene expression^47–50^. In line with this possibility, Brf1 is known to interact with both the C-lobe and positively charged residues of the N-lobe of TBP through an evolutionarily conserved disorder segment. The competition for the same interface by BTAF1/Mot1p, which can evict TBP, could possibly explain why Pol III genes have less TBP turnover^44^.

### The molecular signatures identified are consistent with mutational studies

To assess the importance of the signature positions identified in our study, we performed a comprehensive literature review of previous work describing the effects of mutating different TBP residues. Since TBP has been extensively investigated for over three decades, we identified a number of relevant studies that had mutated several positions and experimentally characterized their impact on DNA binding and transcriptional activity from several model promoters^36,51–55^. Our analysis shows that of the 54 signature positions identified here in total (8 universal signature residues, 29 C-terminal lobe signatures and 17 N-terminal lobe signatures), we could find experimental data for 20 positions (4 universal signatures, 12 C-terminal lobe signatures and 4 N-terminal lobe signatures). It is striking that in each of these cases, mutating the signature positions clearly resulted in reduced DNA binding or transcriptional activity (Supplementary Fig. 9a, b).

We highlight some specific mutational studies below: for the universal signature positions, mutating F116 (N.L5.1) to alanine in yeast and human TBP reduced DNA binding and mutating F305 (C.L5.1) to alanine in human TBP resulted in a significant reduction in transcription in vitro. This is consistent with the importance of these positions in recognizing the DNA. Among the C-terminal lobe positions, E284R (C.L3.2) and E286R (C.L3.4) in human TBP resulted in a significant reduction in transcription at multiple model promoters in vitro. This is consistent with what one might expect for mutations that convert the negatively charged patch to positively charged residues, given their role in mediating interaction with TFIIB. Among the N-terminal lobe signatures, R118E in human TBP results in significant transcription reduction in vitro at one or more model promoters; R235E and R239S in human TBP results in the complete disruption of basal transcription. These observations are consistent with the role of the signature positions in mediating interactions between TBP and the various factors involved in transcriptional initiation. For a complete list of mutations in signature positions and their impact, as well as the mapping of all the mutated positions onto the structure of TBP, please see Supplementary Fig. 9a, b. Taken together, these mutational studies serve as supportive functional validation of the importance of the signature positions that we have identified.

### TBP paralogs in multicellular organisms potentiate the evolution of a new functional repertoire

**Fig. 5:**
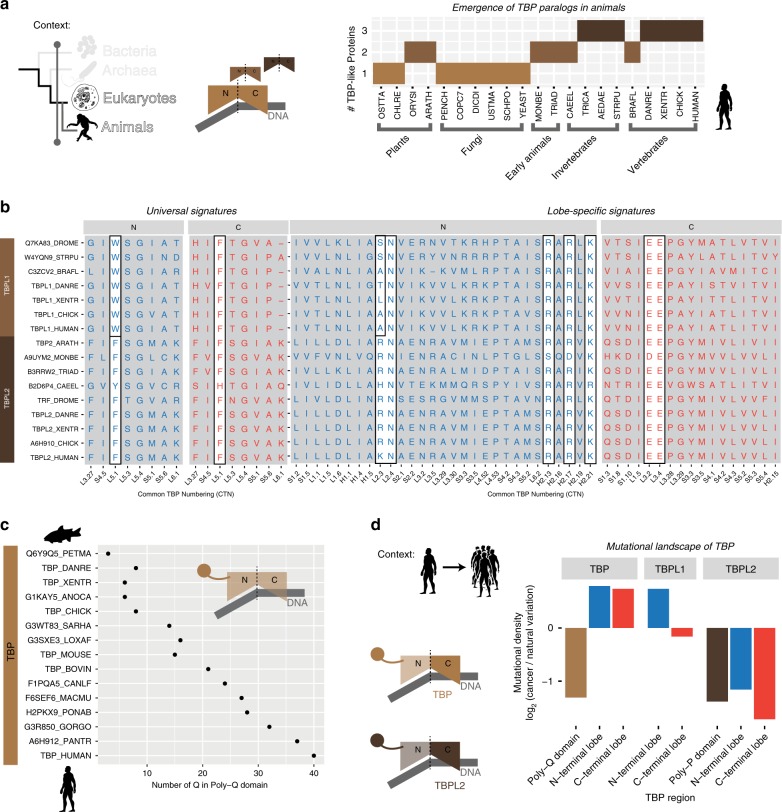
TBP and its paralogs in multicellular species. **a** The spatio-temporal context involves only animals (temporal) and the entire TBP molecule to understand molecular events beyond the sequence of TBP-lobes, i.e. gene duplication and sequence expansion events in animals. The plot (right) displays the number of TBP-like proteins (gene duplicates or paralogs) across various eukaryotic lineages, wherein the *x*-axis contains the UniProt taxonomy code for organisms (https://www.uniprot.org/taxonomy/). **b** The alignment of N-terminal lobe and C-terminal lobe regions of TBP paralogs (i.e. TBPL1 and TBPL2) in animals, highlights the molecular signature positions (universal signatures and lobe-specific signatures) described earlier (boxes). **c** Expansion of PolyGln stretches in N-terminal regions of TBP in terms of the number of Gln residues from different vertebrates, arranged approximately by their evolutionary distances from sea lamprey to humans (*y*-axis is the UniProt ID). **d** Mutational landscape of TBP and its paralogs in the human population for each of the three TBP regions: N-terminal expansion (PolyGln containing region in TBP or PolyPro containing region in TBPL2), N-terminal lobe and C-terminal lobe. The height of the bars represent log ratio of mutational density between cancers and natural variations (see “Methods” section). The positive and negative values of bars indicate relative enrichments in cancers and natural variation, respectively.

Our results so far have revealed that TBP-interacting factors have continuously evolved to interact with TBP, which remained largely invariant at functionally relevant sites. Hence further functional innovation of TBP in eukaryotes would require major molecular transitions. Interestingly we observed the emergence of gene-duplicates of TBP with the advent of multicellularity in eukaryotes (Fig. 5a)^56,57^. The emergence of TBP gene duplicates raises an interesting question as to how the different copies of TBP adapted to more specialized functions, since it seems to coincide with increasing organismal complexity.

To identify the basis of functional diversification of the TBP paralogs^57,58^, we first utilized profile-based sequence searches to identify the organisms where paralogs have emerged (Fig. 5a). We detected at least one TBP paralog in the metazoan species, including the primitive animals (e.g. *Monosiga brevicolis* and *Trichoplax adhaerens*). Most invertebrate and vertebrate genomes contained three TBP paralogs, namely TBP, TBPL1, and TBPL2 (Fig. 5a; Supplementary Data 9). However, primitive animals only contained orthologs of TBP and TBPL2 suggesting that TBPL1 emerged later. We found that TBPL2 is more similar to TBP than TBPL1 (Supplementary Fig. 10a; Supplementary Data 10). Thus although TBPL1 emerged most recently, it appears to have diversified in its sequence with respect to TBP as compared to TBPL2 (Supplementary Fig. 10a); this may cater for more specialized transcriptional regulation^59^. Despite these differences, we found that the TBP paralogs mostly preserve the same set of molecular signature position (i.e. universally conserved, C-terminal and N-terminal lobe signature residues) and are likely to be subject to the same mode of regulation (Fig. 5b). Furthermore, the molecular signature residues of TBP that show gene expression changes or compromised DNA binding when mutated are also conserved in TBP paralogs suggesting their functional relevance. While this may imply that the TBP paralogs do not operate orthogonally to each other, it does not preclude that they may be involved in different functional context as part of distinct transcriptional complexes. In line with this possibility, there are some noteworthy exceptions, with the most striking being TBPL1’s N.L5.1 (TBPL1.N.L5.1) being a Trp rather than Phe. This suggests that perhaps TBPL1 could also inherently recognize specialized DNA motifs and act at a distinct set of promoters.

Beyond the similarity in the core pseudosymmetric architecture of TBP and its paralogs, there are significant differences that stem from large N-terminal expansions. Through profile-based sequence searches we identified that the N-terminus of early vertebrate TBP contains a conserved protein region that encompasses PolyGln residues, which have been suggested to mediate interactions with other proteins and promote the formation of condensates through phase separation (Supplementary Fig. 10b)^60,61^. Interestingly, this Gln repeat increases in length from fish to humans reaching up to ~38 Glns (Fig. 5c; Supplementary Data 11). In humans, expansion beyond the 38 Gln can lead to potential pathological situations^62–64^. Hence there appears to be a balancing selection between the potential utility of Gln repeat and their possible deleterious consequences^60,65,66^. This region is present only in TBP and its vertebrate orthologs; none of the other TBP paralogs have a PolyGln stretch. Similarly, TBPL2 contains a Pro-rich region that is not present in the other TBP paralogs (Supplementary Fig. 10c; Supplementary Data 12). In contrast, TBPL1 does not contain any domain expansion upstream of the N-terminal lobe.

To assess the evolutionary pressure on the new N-terminal expansions and the TBP core, we analyzed (i) natural variation data (missense mutations) from the human population from over 100,000 healthy individuals (gnomAD) and (ii) somatic cancer mutation data (cBioPortal database^67^) by mapping them onto TBP and its paralogs. Using this data, we calculated the enrichment between cancer mutations and natural variations for the different regions (Fig. 5d). We found a high enrichment for natural variation in the PolyGln region of TBP. However for both the N-terminal and C-terminal lobes, there is an appreciable enrichment of cancer mutations as compared to natural variations. This suggests that the missense mutational rates in the PolyGln-containing region is higher in the human population and at the same time less variable in cancer genomes (Fig. 5d). Consistent with this observation, we also observed that naturally occurring mutations, such as indels or frameshifts are more prominent in this region than the rest of the protein (see Supplementary Data 13). Indeed, it has also been observed that TBP PolyGln repeat length varied between individuals and ranges from 25 to 42 repeats in the human population^68^.

An investigation of large-scale datasets on human protein–protein interactions revealed that about half of the interaction partners of TBPL1 and TBPL2 (11/23 and 4/8, respectively) overlap with TBP (Supplementary Fig. 10d). Notwithstanding knowledge bias associated with more studies on TBP compared to its paralogs, this indicates a substantial overlap in functionality, but also significant neo-functionalization via the emergence of novel interactions that are distinct for the paralogs. Examination of the human tissue-specific protein expression profiles (Supplementary Fig. 10e) revealed a relatively high correlation in protein expression for human TBP with TBPL2 (~0.7) and a much lower correlation with TBPL1 (~0.2). Furthermore, TBPL1 seems to be more ubiquitously expressed across tissues, particularly in fetal tissues and tissues involved in reproduction. Taken together, these observations collectively suggest that differences in signature residues and differential N-terminal expansions, which may act as potential interaction mediating modules, as well as divergence in the expression pattern of TBP and its paralogs may have contributed to functional diversification and specialization of TBP and its paralogs whilst keeping the core biochemical functionality intact^59,69–71^.

## Discussion

**Fig. 6:**
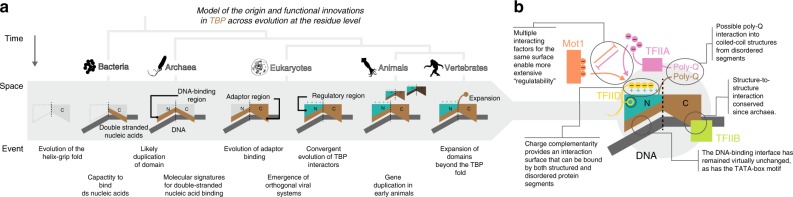
Unified, multi-stage model for the functional innovations of TBP. **a** Evolutionary model for the functional innovations of TBP from bacteria to vertebrates. This includes the emergence of orthogonal TBP–TFIIB system in viruses. The functional innovations range from binding double-stranded nucleic acids by a simple, single TBP-lobe unit in bacteria to becoming a hub for complex gene-expression regulation and functional diversification via TBP paralogs and N-terminal expansions in vertebrates. **b** Functional innovations of TBP enable it to integrate distinct regulatory signals in eukaryotes.

We have investigated key molecular characteristics of TBP in various spatio-temporal contexts and identified signature positions for the major functions by analyzing sequences that emerged at different times: from millions of years of evolution across the superkingdoms of life to thousands of years of evolution within the human population. We have also explored how these signatures are exploited by various TBP-interacting factors that emerged at different evolutionary branch points. This allowed us to arrive at a unified multi-stage model of the sequence of functional innovations of TBP (Fig. 6a, Supplementary Fig. 11).

The universally conserved signature positions of TBP are predominantly involved in double-stranded nucleic acid binding, which is the fundamental function for most of the known members of the TBP family. Specifically, the highly conserved aromatic residues at L5.1 could contribute significantly towards double-stranded nucleic acid binding through a common mode of interactions. At the advent of archaea, TBP most likely emerged after a duplication event from a bacterial precursor^37^ and has fine-tuned DNA interaction through other DNA-binding residues. The conserved Phe at L5.1 that is present in most archaeal, eukaryotic, and viral TBPs, makes contacts with TA sequences. Hence, the duplication and fusion events likely contributed to the recognition of TATA-box sequences, enabling TBP to mark sites for transcription initiation.

The emergence of an adapter module (TFB) in the C-terminal lobe occurred in archaea and facilitated the recruitment of the polymerase for transcription initiation. The presence of signature positions mediating interaction with TFIIB and its paralogs (with the exception of TAF1B) in humans suggests that the mode of adapter recruitment is likely to be preserved. This might be mediated via a salt bridge and electrostatic interaction formed by positions C.L3.2 and C.L3.4, respectively. Because of its distal position to the C-terminally located adapter domain, the N-terminal lobe offers a suitable interface where regulation of transcription can happen. The signature positions in the N-terminal lobe (i.e. regulation domain) mediate interactions with various regulatory factors that may have opposing effects on transcriptional output (e.g. BTAF1/Mot1p evicting TBP from the promoter and TFIIA stabilizing it). The different regulatory proteins are evolutionarily unrelated yet make contacts with the same interface region on the N-terminal lobe. They do so through dynamic protein segments that are enriched in negatively charged residues, interspersed with aromatic residues. Given the convergent nature of the region that mediates interaction with the N-terminal lobe of TBP, it is likely that multiple other factors (e.g. Myc and Med8; Supplementary Data 14 and 15) containing such segments can bind TBP in a similar manner^72^. Furthermore, earlier mutation studies of TBP offer a supporting functional validation of the importance of many of the signature positions identified through our approach.

A common theme that unites various functional innovations of TBP is that there has been evolutionary pressure on TBP to present highly similar core set of interacting residues over a wide phylogenetic range and that this was exploited differently by distinct TBP-interacting factors during the course of evolution. In eukaryotes, this played out on at least on two occasions: (i) during the emergence of paralogous transcription systems (Pol II and III), where the divergent evolution of factors maintained an overall similar mode of interaction with the C-lobe of TBP and (ii) during the emergence of the regulatory region within the N-lobe of TBP, where diverse and numerous seemingly unrelated factors have evolved in a convergent manner to interact with the same TBP interface positions. The latter event suggests that interactions mediated by disordered regions are an effective means for convergent evolution.

Despite the evolutionary pressure on TBP, the emergence of TBP paralogs appears to have unlocked the ability for more rapid evolution. Indeed, several signature positions appear to have diverged in one of the TBP paralogs. For instance, N.L5.1, which is a universally conserved position (Phe) that contacts the TATA-box has been systematically conserved as a Trp at the N-terminal lobe in TBPL1, suggesting that this paralog could bind to a different promoter sequence. Furthermore, in vertebrates PolyGln and Proline-rich regions have evolved in TBP and TBPL2, respectively, which could mediate new protein–protein interactions^73–75^. Thus, the emergence of gene duplicates has enabled the exploration of new functional landscapes in TBP and each of its paralogs.

It is interesting to note that the viral TBP sequences still maintain the signature positions important for double-stranded nucleic acid binding. However, the signature positions for interacting with the adapter and the host regulatory proteins have diverged. This implies that the viral TBP sequences are unlikely to recruit host TFIIB and potentially evade regulation by the host factors. Consistent with this, several viruses appear to have evolved their own TFIIB homologs. Moreover, the signature positions on the viral TBP sequence and the cognate TFIIB sequence appear to have coevolved across different viruses. These observations suggest that viral TBP and TFIIB have the potential to operate as independent orthogonal transcription systems. The diversity of viral TBP-like and TFIIB-like proteins described here could have implications for synthetic biology applications for manipulating transcription in diverse eukaryotic systems. It should be noted that as viruses evolve their sequences more rapidly, it still might be possible for them to interact with coactivators through a distinct mode.

In summary, the evolution of TBP reflects a delicate balancing act, where core functional characteristics are preserved while allowing the emergence of novel functional features (Fig. 6b). Recent advances towards complete identification of transcription complexes in atomic detail could provide more insights into understanding the interactions of TBP with other factors in diverse contexts. This might help elucidate the functions of some of the signature positions that are yet to be linked to specific functions, as well as reveal lineage-specific adaptations of this key protein involved in transcription. In this context, the signature positions identified here can be used to design new experiments to probe TBP function. We hope that the principles and findings described in this work will provide a framework not only for understanding TBP but also more generally to help investigate and understand the roles of various central interaction “hub” proteins that are conserved across a wide phylogenetic range.

## Methods

### Construction of RefMSA

TBP lobe alignment and TBP common numbering scheme: We sought to build a Reference Multiple Sequence Alignment or RefMSA that would contain representative TBP-lobe sequences from major lineages in three superkingdoms of life. This RefMSA would facilitate various comparative analyses without the requirement of a reference protein structure. As the first step towards the generation of RefMSA, we sought to build a structure-based sequence alignment of known TBP structures (see Supplementary Fig. 12 and Supplementary Data 16). Towards this goal, we adopted the following steps: (1) we collected all the known structures of TBP in archaea and eukaryotes at atomic details from the PDB database (www.rcsb.org; https://www.ebi.ac.uk/pdbe/). (2) We then processed this data to obtain non-redundant structures such that only one structure per organism is selected, with the preference being given to the structure with the best crystallographic resolution in a given organism. (3) We then separated TBP structures into “TBP lobes” (N-terminal and C-terminal lobe) based on visual inspection of structures in PyMOL (The PyMOL Molecular Graphics System, Version 2.0 Schrödinger, LLC). (4) Finally, we created a structure-based sequence alignment (multiple sequence alignment) of these separated TBP lobes using MUSTANG^76^. We also overlaid the assigned DSSP secondary structural information for each TBP lobe in the above alignment to arrive at the common TBP numbering scheme based on consensus secondary structural elements (see below for more details).

Identification of TBP orthologs and homologs: The above structure-based sequence alignment was used to search the entire UniProt and TrEMBL databases^77^ through profile-based sequence search software such as JACKHMMER and PSI-BLAST^78–80^. These searches enabled us to identify archaeal and eukaryotic orthologs and homologs (Supplementary Fig. 12). The identified orthologs and homologs were independently confirmed by performing the above sequence search procedure using the respective full length TBP sequences in the structure-based sequence alignment as queries. For all the sequence searches, we set an *e*-value threshold of 0.001 for best hits (reciprocal best hits with the human TBP sequence was used to define orthologs). Only sequence hits that covered at least 70% of query lengths were considered. These hits were also independently confirmed by consulting the data available in the Pfam and OMA databases^81,82^.

Selection of TBP lobe sequences for RefMSA: To ensure a homogenous and balanced representation from every major lineage of eukaryotes and archaea, we only considered hits (orthologs at TBP lobe level) from two representative organisms from each major lineage (e.g., chordates, SAR group, fungi, euryarchaeota, crenarchaeota, etc.; in consultation with NCBI Taxonomy database^83^; see Supplementary Data 1). Once the representatives from lineages were selected, the regions of these representative sequence hits that matched to the query alignment were aligned to the query structure-based sequence alignment using HMMER profiles^78^ without altering the original alignment as much as possible. By this process, we arrived at a more comprehensive sequence alignment spanning a diverse range of eukaryotic and archaeal lineages that cover significant diversity. We refer to this as the initial RefMSA.

Distant homologs of TBP lobes and final RefMSA: We then identified distant homologs of the TBP lobes represented in the initial RefMSA by providing the initial RefMSA as a query to the following profile-based sequence search procedure (Supplementary Fig. 12). This procedure involved: (i) iterative JACKHMMER searches performed on combined UniProt and TrEMBL databases (*e*-value cut-off = 0.001 and query length cut-off = 60%) and (ii) HHPred searches^84,85^ (probability cut-off = 75%) performed on PDB and Pfam databases. The entire sequence search procedure yielded distant bacterial homologs, such as RNase HIII and rnlA toxins hits as well as viral TBP homologs. Based on the availability of crystal structures of the above search hits, we also performed structural searches using DALI and Topsearch servers^86,87^ and visual inspections using UCSF Chimera to confirm the homology^88^. The distant homologs of TBP lobes such as RNase HIII and some viral TBP homologs were already identified^37^ (UniProt database). However, to the best of our knowledge, rnlA toxins and poxviruses TBP homologs are novel identifications. We then integrated representative sequences of the distant homologs from major lineages in bacteria and viruses, including all the homologs in poxviruses. We termed this comprehensive alignment as RefMSA. While we included TBP paralog lobe sequences in animals in the RefMSA, TBP paralogs were not considered for the ancestral sequence reconstruction towards identification of universally conserved molecular signatures and for the C-terminal and N-terminal lobe-specific signatures. During the course of distant homolog searches we identified single lobe versions of TBP in archaea, consistent with an earlier study^37^.

### CTN scheme

As mentioned above, we developed a CTN system by integrating secondary structure information from available crystal structures of TBP lobes with the initial structure-based sequence alignment. This allowed us to decipher consensus secondary structure for each residue position in the alignment (see above and Supplementary Fig. 2a). This also enabled us to uniquely assign an alignment position to a combination of three types of information: (1) lobe—N-terminal or C-terminal lobe. (2) Secondary structural element, i.e. “H” for helix, “S” for strand and “L” for loop, along with the index of the secondary structural element, i.e. “H1” stands for helix number 1 and “S2” stands for strand number 2, etc. (3) Residue number of the alignment position within the index of the given structural element, i.e. “H1.12” denotes 12th position in helix number 1, “S4.3” denotes 3rd position in strand number 4, L3.2 denotes 2nd position in loop number 3, etc. For instance, C.L3.2 refers to the second position in loop L3 in the C-terminal lobe of TBP (Supplementary Fig. 2a). We then mapped this information on to the RefMSA to arrive at the final CTN numbering for the RefMSA.

### Universally conserved residues in TBP lobes

Ancestral sequence reconstruction for superkingdoms: The number of TBP lobe sequences in RefMSA differs between the three superkingdoms (i.e. archaea, bacteria, and eukaryotes). Hence, in order to decipher critical residues in a biological meaningful way, we devised an ancestral sequence reconstruction strategy for each superkingdom. The ancestral sequence reconstruction method aims to determine the most probable ancestral sequence for every superkingdom to aid meaningful comparison across the three superkingdoms. From the RefMSA, we extracted superkingdom-specific sequence alignments of TBP lobes for each superkingdom, i.e. for archaea, eukaryotes, and bacteria. Then the ancestral sequence for each of the superkingdom was deduced by running the maximum-likelihood encoding program FASTML^89^ (http://fastml.tau.ac.il/) on each of the superkingdom-specific alignments (Supplementary Fig. 12). This process yielded an ancestral sequence for each superkingdom with a sequence length of 209 residue positions (including gaps), exactly the number of alignment positions in RefMSA. Hence, the AncesRefMSA acronym for ancestral RefMSA contained three sequences with 209 alignment positions (Supplementary Fig. 12). As each position in the ancestral sequence corresponds to a respective alignment position in the RefMSA, the CTN of the RefMSA (see the Results) also applied to any position in the AncesRefMSA as well.

### Identification of universally conserved positions

We then evaluated highly conserved positions in the AncesRefMSA, which are considered as universally conserved positions, through the following steps:

(i) Calculate normalized BLOSUM (BLOSUM62) score for each position in the AncesRefMSA as follows:

- For any given alignment position *n* and amino acid residues at this position for three superkingdoms being *R*_*i*_, where *i* = 1,2,3 {1 = archaea, 2 = eukaryotes, and 3 = bacteria}. We evaluated normalized BLOSUM score at a given alignment position “*n*” (NBS_*n*_)*:*

1 $${\mathrm{NBS}}_{n} = \left[ {{\mathrm{\Sigma }}_{{i} = 1,2}{\mathrm{\Sigma }}_{{i} \ne {j},{j} = 2,3}\;{\mathrm{BS}}_{{ij}}} \right]/3{\mathrm{C}}_2$$

where BS_*ij*_ = BLOSUM score (*R*_*i*_ → *R*_*j*_)/Maximum [BLOSUM score (*R*_*i*_ → *R*_*i*_), BLOSUM score (*R*_*j*_ → *R*_*j*_)] and “ →“ refers to amino-acid residue substitution, NBS_*n*_ values would be in the interval [−1,1]. “C” represents combinatorial symbol, where as *n*C_*m*_ = *n*! /[(*n*−*m*)! * *m*!], “!” denotes factorial.

(i) Evaluate mean and standard deviation of all NBS_*n*_ over all the 209 positions in AncesRefMSA:
  2 $${\mathrm{Mean}}\,{\mathrm{NBS}} = \left[ {\mathop {\sum}\nolimits_{n = 1..209}{{\mathrm{NBS}}_{n}}}\right]/209$$
  SDNBS is the standard deviation of all NBS_*n*_ over 209 positions.
(ii) For any given alignment “*n*”, NBS_*n*_ > (Mean NBS + SDNBS) denotes a “Universally conserved position” or a highly conserved position.

By this procedure we identified eight such positions, out of which five positions map to double-stranded nucleic acid contacting residues in known crystal structures. These universally conserved positions were spatially distributed on TBP lobes, which define the molecular signature for double-stranded nucleic acid binding.

### Molecular signatures in C-terminal and N-terminal lobes

We developed a distinct strategy that would simultaneously incorporate spatio-temporal contexts for the identification of these molecular signatures specific for N-terminal and C-terminal lobes (Supplementary Fig. 12). This is motivated by the fact that: (a) sequence homology within archaeal and eukaryotic TBP lobe sequences from RefMSA is significantly closer than when we included TBP-like sequences of bacteria and (b) there is an identical number of N-terminal and C-terminal lobe sequences (pseudo-symmetry in TBP) in archaeal and eukaryotic lineages (equal representation).

C-terminal or N-terminal lobe-specific signatures were calculated as follows (Supplementary Fig. 12):

(i) For a given spatio-temporal context (i.e. Archaea and Eukaryotes for C-terminal lobe signature identification or Eukaryotes only for N-terminal lobe signature identification), we first segregated two sets of alignment namely the TBP C-terminal and N-terminal lobe alignments from RefMSA. Both these set of alignments will have the same number of alignment positions (Supplementary Fig. 12).
(ii) We evaluated normalized BLOSUM (BLOSUM62) scores for each alignment position and also mean normalized BLOSUM scores in both the alignments independently as follows:

- For any given alignment position “*n*” in both the alignments (C-terminal or N-terminal lobe alignments), amino acid residues at this position being NR_*i*_ for the N-terminal lobe alignment and CR_*i*_ for the C-terminal lobe alignment, where *i* = 1 to *m*. “*m*” is the total number of TBP lobe sequences in each of the alignment.
  Calculate normalized BLOSUM score for C-terminal or N-terminal lobe alignment (NBS_*n*_) = $\left[ {\mathop {\sum}\nolimits_{{i} = 1..{m} - 1} {\mathop {\sum}\nolimits_{{i} \ne {j},\;{j} = 2..{m}} {{\mathrm{BS}}_{{ij}}} } } \right]/m{\mathrm{C}}_2$
  where for the N-terminal lobe alignment: BS_*ij*_ = BLOSUM score (NR_*i*_ → NR_*j*_)/Maximum [BLOSUM score (NR_*i*_ → NR_*i*_), BLOSUM score (NR_*j*_ → NR_*j*_)] and “ →“ refers to amino-acid residue substitution.
  Similarly, for C-terminal lobe alignment: BS_*ij*_ = BLOSUM score (CR_*i*_ → CR_*j*_)/Maximum [BLOSUM score (CR_*i*_ → CR_*i*_), BLOSUM score (CR_*j*_ → CR_*j*_)]
- Evaluate the mean of all NBS_*n*_ over all the 209 positions for C-terminal and N-terminal lobe groups separately:

Mean NBS (for both C-terminal and N-terminal lobes) = $\left[ {\mathop {\sum}\nolimits_{n = 1..209} {{\mathrm{NBS}}_{n}} } \right]/209$

(i) C-terminal lobe specific signature: “*n*” is a given alignment position in the RefMSA. If NBS_*n*_ (C-terminal lobe) > Mean NBS for C-terminal lobe and NBS_*n*_ (C-terminal lobe) > 1.5 * NBS_*n*_ (N-terminal lobe), then this alignment position is a part of the molecular signature positions. However, in cases if NBS_*n*_ (C-terminal lobe) > Mean NBS for C-terminal lobe and NBS_*n*_ (C-terminal lobe) ≤ 1.5*NBS_*n*_ (N-terminal lobe). We sought to find if the most conserved residue at the alignment position “*n*” in C-terminal lobe alignment is different from the most conserved residue at the same alignment position “*n*” in the N-terminal lobe alignment. If this indeed the case, then such a position is still considered to be part of the molecular signature for the C-terminal lobe.
(ii) N-terminal lobe-specific signature: “*p*” is a given alignment position in the RefMSA. If NBS_*p*_ (N-terminal lobe) > Mean NBS for N-terminal lobe and NBS_*p*_ (N-terminal lobe) > 1.5*NBS_*p*_ (C-terminal lobe), then this alignment position is a part of the molecular signature positions. However, if NBS_*p*_ (N-terminal lobe) > Mean NBS for N-terminal lobe and NBS_*p*_ (N-terminal lobe) ≤ 1.5*NBS_*p*_ (C-terminal lobe). We sought to find if the most conserved residue at the alignment position “*p*” in the N-terminal lobe alignment group is different from most conserved residue at the same alignment position “*p*” in the C-terminal lobe group. If this is the case then such a position is still considered to be part of the molecular signature for the N-terminal lobe.

### Alignment of TBP-interacting factors

Eukaryotic orthologs of human BTAF1/yeast Mot1p, human TAF1B/yeast Rrn7p, human BRF1/yeast Brf1p, yeast Toa1p and Toa2p, human BRF2 and TFIID (TAF1) were identified using profile-based sequence searches, such as JACKHMMER and PSI-BLAST performed on entire Uniprot and TrEMBL databases. The ortholog sequence hits or matches were also confirmed by consulting data in the Pfam, OMA, and Ensembl databases. Archaeal orthologs as well as viral homologs of human TFIIB (GTF2B) were identified using the above sequence search strategy. We only considered a representative from each of the major lineages of eukaryotes, archaea, and viruses. For each of the TBP-interacting factor, we constructed a multiple sequence alignment of its orthologs using MSAPROBs^90^ and HMMER profiles spanning the regions of interactions. These alignments were further manually corrected, based on secondary structure assignment of the representative structure(s) and Jpred^91^ predictions of secondary structural regions.

### Repertoire of TBP-like and TFIIB-like sequences in viral genomes

We detected TBP-like sequences in dsDNA virus genomes using the intial RefMSA as a query for JACKHMMER/PSI-BLAST searches on viral sequences data in UniProt and TrEMBL databases (Supplementary Figure 12; see above). We constructed a multiple sequence alignment using MSAPROBs and HMMER profiles of TFIIB and its homologs in eukaryotes and archaea (see above). Using this alignment as a query for JACKHMMER/PSI-BLAST searches, we identified TFIIB-like sequence in dsDNA viral genomes. Using the above sequence search approach, we were able to detect TBP-like sequences and TFIIB-like sequences in 107 and 129 viral genomes, respectively. However, we were not able to identify any TFIIB homolog in poxviruses.

### Co-evolution in viral TBP–TFIIB interaction interface

Integration of viral TBP and TFIIB alignment: TBP sequences in dsDNA viruses that span the entire pseudo-symmetric structure were obtained from the RefMSA. A multiple sequence alignment of the above viral TBP sequences was constructed using HMMER profiles and MSAPROBs. Viral orthologs of eukaryotic TFIIBs were obtained using profile-based sequence searches (see Supplementary Fig. 12 and above). We constructed the alignment of viral TFIIBs orthologs using HMMER profiles and MSAPROBs. We also integrated the viral TBP and TFIIB alignments together as a single alignment by appending or juxtaposing one alignment to another and ensuring that the viral TBP and TFIIB are from the same viral species (Supplementary Data 6; Supplementary Fig. 12). Interestingly, we could not identify any TFIIB-like protein in poxvirus genomes and therefore poxviruses were not further considered.

Estimation of co-evolution in viral TBP–TFIIB interaction sites: Using the above viral TBP–TFIIB integrated alignment, we evaluated the extent of co-evolution of all the positions in the viral TBP with viral TFIIB as follows, using the Pearson correlation as a measure of co-evolution that has been used in numerous earlier studies:

- Firstly, Pearson correlation of the BLOSUM scores (see below) for residue substitutions of any given alignment position in viral TBP alignment and of all positions in viral TFIIB alignment was calculated (Supplementary Fig. 12 and Supplementary Data 6). We particularly focused on co-evolution of the alignment positions in the viral TBP alignment that are equivalent to CTN positions C.L3.2 and C.L3.4 in the RefMSA and their inferred interaction sites in the viral TFIIBs (based on available crystal structure data on eukaryotic TBP–TFIIB interactions).
  The correlation in residue substitutions was evaluated as Pearson correlation (PC) in amino-acid substitution similarities across sequences in the alignment between: (i) a given alignment position (denoted by r) in viral TBP and (ii) another given alignment position in viral TFIIBs (denoted by s) (Supplementary Fig. 12).
  All pairwise BLOSUM substitution scores at the alignment position *r*:
  *B*_r_ = {BS_*i*=1..*n*−1j≠i, *j*=2..*n*_}, where BS_*ij*_ = BLOSUM score and (*R*_*i*_ → *R*_*j*_) amino acid residue substitution at position r between sequence “*i*” and sequence “*j*”
  All pairwise BLOSUM substitution scores at the alignment position *s*:
  *B*_s_={${\mathrm{BS}}\prime _{{i} = 1..{n} - 1{i} \ne {j},{j} = 2..{n}}$}, where ${\mathrm{BS}}\prime _{{ij}}$ = BLOSUM score and $\left( {{R}\prime _{i} \to {R}\prime _{j}} \right)$ amino acid residue substitution at position s between sequence “*i*” and sequence “*j*”
  “*n*” is the total number of sequences in the alignment, hence the total number of entries in *B*_r_ or *B*_s_would be *n*C_2_.
  $${\mathrm{Pearson}} \, {\mathrm{correlation}} \, {\mathrm{coefficient}} \, {\mathrm{(PC)}} \, = \, \frac{{\left[ {\left( {{N} \ast {\mathrm{\Sigma }}_{{i} = 1..{N}}\quad {B}_{\mathrm{r}}{B}_{\mathrm{s}}} \right) - \left[ {\left( {{\mathrm{\Sigma }}_{{i} = 1..{N}}\quad {B}_{\mathrm{r}}} \right) \ast \left( {{\mathrm{\Sigma }}_{{i} = 1..{N}}\quad {B}_{\mathrm{s}}} \right)} \right]} \right]}}{{\sqrt{ \left[ {\left( {{N} \ast {\mathrm{\Sigma }}_{{i} = 1..{N}}{B}_{\mathrm{r}}^2} \right) - \left( {{\mathrm{\Sigma }}_{{i} = 1..{N}}{B}_{\mathrm{r}}} \right)^2} \right] \ast \left[ {\left( {{N} \ast {\mathrm{\Sigma }}_{{i} = 1..{N}}{B}_{\mathrm{s}}^2} \right) - \left( {{\mathrm{\Sigma }}_{{i} = 1..{N}}{B}_{\mathrm{s}}} \right)^2} \right]}}}$$
  where *N* = *n*C_2_ = total number of elements in *B*_r_ or *B*_s_
- Next randomization of amino acids at a given position in the viral TBPs (alignment position r) and its inferred interaction site (alignment positions) at viral TFIIB were done independently. But at the each site in the alignment we maintained the original amino-acid distribution during randomization. We then evaluated *B*_r_, *B*_s_ and the corresponding Pearson correlation coefficient (PC-random) for these randomized sites as in the above step a). This randomization procedure and evaluation of PC-random was repeated for 1000 times. The *p*-value was computed as the number of times the PC-random was greater than or equal to the original PC (in step a)).

### Inter-molecular and intra-molecular interaction mediating residues

Inter-molecular and intra-molecular residue to residue contacts or interactions were determined using van der Waals contacts between atoms as described in Venkatakrishnan et al. ^92^ and Kayikci et al. ^93^. Inter-molecular residue-to-residue interactions were also confirmed using the data available in PDBe-KB (https://www.ebi.ac.uk/pdbe/pdbe-kb)^94^.

### Calculation of disorder propensities

Evaluation of the propensity for interaction mediating regions of TBP-interacting proteins to be disordered was done using IUPred^95^. If the disorder propensity value >0.5 for a given residue then it was considered to be in a disordered region. The output of disorder propensities for each sequence in the respective alignments was plotted against the amino-acid residue numbers and the average and standard deviation were also estimated and indicated in the plot.

### Human natural variation and cancer mutation data

Natural variation data for missense mutations and other mutations such as frameshifts and indels (insertions and deletions) was downloaded from the gnomAD database (https://gnomad.broadinstitute.org/), which contains a comprehensive collection of exome and genome-sequencing data from a wide range of large-scale-sequencing projects. Cancer mutation data was downloaded from the CBioPortal (https://www.cbioportal.org/)^67^. These datasets were utilized to obtain natural variation data for TBP and its interaction factors (based on Supplementary Data 1 and 7). Mutation density (MD) for a given region or domain of interest was calculated as follows:

$${\mathrm{MD}} = \frac{{{\mathrm{No.}}\;{\mathrm{of}}\;{\mathrm{missense}}\;{\mathrm{mutations}}}}{{{\mathrm{Sequence}}\;{\mathrm{length}}\;{\mathrm{of}}\;{\mathrm{the}}\;{\mathrm{region}}\;{\mathrm{or}}\;{\mathrm{domain}}}}$$

The mutational density-enrichment ratio (MDR) was calculated for a given region or a domain of interest as

$${\mathrm{MDR}} = {\mathrm{log}}_2\left( {\frac{{{\mathrm{MD}}\left( {{\mathrm{for}}\;{\mathrm{cancer}}\;{\mathrm{mutations}}} \right)}}{{{\mathrm{MD}}\left( {{\mathrm{for}}\;{\mathrm{natural}}\;{\mathrm{variation}}} \right)}}} \right)$$

### Identification of PolyGln and proline-rich regions

Homologs of human PolyGln-containing and Proline-rich region sequence segments were identified using human TBP (UniProt ID: TBP_HUMAN, residues 1–163) and human TBPL2 (UniProt ID: TBPL2_HUMAN, residues 1–200), respectively, as a query to JACKHMMER searches. The searches performed on the whole UniProt database with an *e*-value cut-off of 0.001. We then considered hits with a match of at least 60% sequence coverage to their respective query sequences. We constructed an initial sequence alignment using MSAPROBS and this was further refined using HMMER profiles to arrive at the final alignments (see Supplementary Data 11 and 12). Conserved regions in the final alignment confirmed the existence of evolutionarily conserved positions in the N-terminal regions of TBP and TBPL2 in vertebrates.

### Proteomics data

Protein expression data across 30 different tissues were obtained from http://www.humanproteomemap.org/^96^. Correlation calculations were performed using custom codes written in the R programming language.

### Interaction data

Protein–protein interaction data for TBP, TBPL1, and TBPL2 were obtained from the BIOGRID and INTact databases^97,98^. The Venn diagram was generated using the website http://bioinformatics.psb.ugent.be/webtools/Venn/.

### Dendrogram construction

For the dendrogram describing the TBP lobe level relationship (Supplementary Fig. 2), an initial approximate maximum-likelihood dendrogram was constructed using the RefMSA, excluding the poxviral TBP lobes. The initial dendrogram was constructed using FastTree^99^, was used as a starting point for the construction of final maximum-likelihood dendrogram using the MEGA7 package^100^ with WAG (g+I) parameters. The dendrogram representation was made using the iTOL database (https://itol.embl.de/)^101^.

For the dendrogram describing the TBP paralogs relationship (Supplementary Fig. 9), an initial approximate maximum-likelihood dendrogram was constructed using FastTree^99^ for TBP-like proteins, including TBP, based on the RefMSA at the full pseudo-symmetric architecture (that include both N-terminal and C-terminal lobes together). Final dendrograms in the figure were made the using MEGA7 package^100^ with WAG (g+I) parameters. The dendrogram representation was made using the iTOL database^101^.

### Reporting summary

Further information on research design is available in the Nature Research Reporting Summary linked to this article.

## Supplementary information

Supplementary Information

Reporting Summary

Description of Additional Supplementary Files

Supplementary Data 1-16

## Data Availability

The data that support this study are available from the corresponding authors upon reasonable request. The supplementary data is available at https://www.mrc-lmb.cam.ac.uk/genomes/tbp/ and provided as a zipped supplementary data file.
