# Supplementary material for: Molecular determinants underlying functional innovations of TBP and their impact on transcription initiation
Source: Nat Commun. 2020 May 13;11:2384. doi: 10.1038/s41467-020-16182-z (PMC7221094; doi:10.1038/s41467-020-16182-z)
Supplement: Supplementary file 1 — Supplementary Information [file 41467_2020_16182_MOESM1_ESM.pdf]

Supplementary Information

# Molecular determinants underlying functional innovations of TBP and their impact on transcription initiation

Ravarani et al.

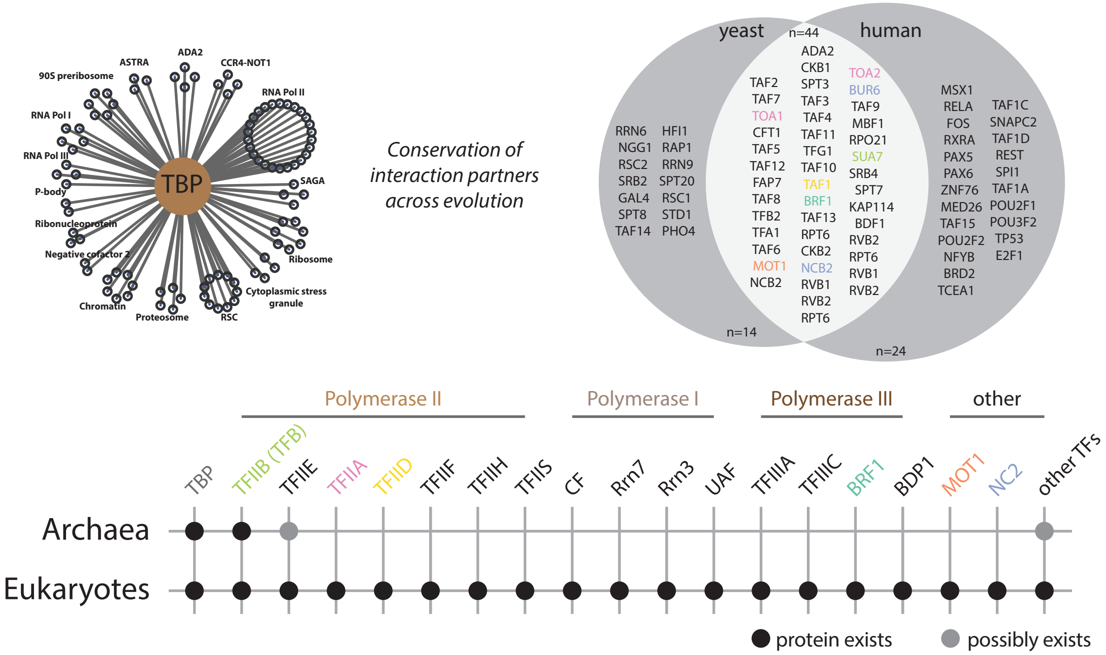

ASTRA
ADA2
CCR4-NOT1
90S preribosome
RNA Pol II
RNA Pol I
RNA Pol III
SAGA
P-body
TBP
Ribonucleoprotein
Negative cofactor 2
Ribosome
Cytoplasmic stress granule
Chromatin
Proteosome
RSC
Conservation of interaction partners across evolution
yeast
human
n=44
RRN6 HFI1
NGG1 RAP1
RSC2 RRN9
SRB2 SPT20
GAL4 RSC1
SPT8 STD1
TAF14 PHO4
n=14
TAF2
TAF7
TOA1
CFT1
TAF5
TAF12
FAP7
TAF8
TFB2
TFA1
TAF6
MOT1
NCB2
ADA2
CKB1
SPT3
TAF3
TAF4
TAF11
TFG1
TAF10
TAF1
BRF1
TAF13
RPT6
CKB2
NCB2
RVB1
RVB2
RPT6
TOA2
BUR6
TAF9
MBF1
RPO21
SUA7
SRB4
SPT7
KAP114
BDF1
RVB2
RPT6
RVB1
RVB2
MSX1 TAF1C
RELA SNAPC2
FOS TAF1D
RXRA REST
PAX5 SPI1
PAX6 TAF1A
ZNF76 POU2F1
MED26 POU3F2
TAF15 TP53
POU2F2 E2F1
NFYB
BRD2
TCEA1
n=24
Polymerase II
Polymerase I
Polymerase III
other
TBP
TFIIB (TFB)
TFIIE
TFIIA
TFIID
TFIIF
TFIIH
TFIIS
CF
Rrn7
Rrn3
UAF
TFIIIA
TFIIIC
BRF1
BDP1
MOT1
NC2
other TFs
Archaea
Eukaryotes
protein exists
possibly exists

**Supplementary Figure 1. Physical interaction partners of TBP and their conservation between organisms**

a) TBP functions as a hub of physical interactions for various transcriptional protein complexes. Some of these protein complexes with TBP include several chromatin-remodeling complexes such as ASTRA, ADA2 and SAGA complexes, basal transcription apparatus such as RNA Pol I, II and III holoenzymes and RNA processing machineries such as CCR4-NOT1.

b) Numerous components of such transcriptional complexes are conserved between organisms and also physically interact with TBP. For instance, about 65% of interaction partners are conserved and physically interact with their respective TBPs between yeast and human TBPs.

c) Basal transcription apparatus and its components that are common between two superkingdoms, i.e. archaea and eukaryotes, are shown. The presence of numerous additional components in eukaryotic basal transcription machinery is possibly due to the existence of parallel transcription systems, i.e. PolI, PolII and PolIII and complex chromatin dynamics during the transcription process.

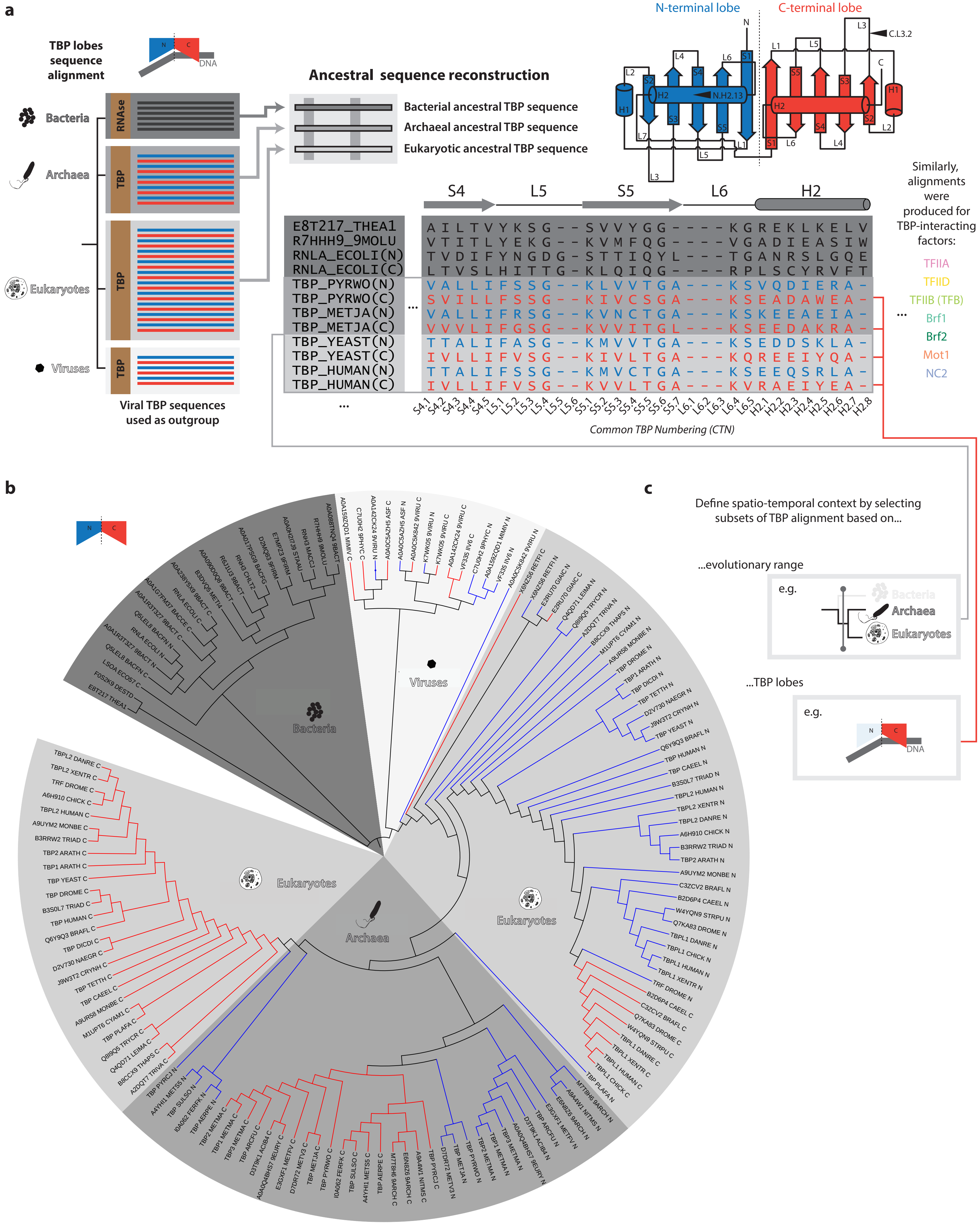
a
TBP lobes sequence alignment
N
C
DNA
Bacteria
Archaea
Eukaryotes
Viruses
RNAse
TBP
Viral TBP sequences used as outgroup
Ancestral sequence reconstruction
Bacterial ancestral TBP sequence
Archaeal ancestral TBP sequence
Eukaryotic ancestral TBP sequence
N-terminal lobe
C-terminal lobe
C.L3.2
N.H2.13
H1
H2
S1
S2
S3
S4
S5
L1
L2
L3
L4
L5
L6
L7
S4
L5
S5
L6
H2
E8T217_THEA1 AILTVYKSG--SVVYGG----KGREKLKELV
R7HHH9_9MOLU VTITLYEKG--KVMFQG----VGADIEASIW
RNLA_ECOLI(N) TVDIFYNGDG-STTIQYL---TGANRSLGQE
RNLA_ECOLI(C) LTVSLHITTG-KLQIQG----RPLSCYRVFT
TBP_PYRWO(N) VALLIFSSG--KLVVTGA---KSVQDIERA-
TBP_PYRWO(C) SVILLFSSG--KIVCSGA---KSEADAWEA-
TBP_METJA(N) VALLIFRSG--KVNCTGA---KSKEEAEIA-
TBP_METJA(C) VVVLIFGSG--KVVITGL---KSEEDAKRA-
TBP_YEAST(N) TTALIFASG--KMVVTGA---KSEDDSKLA-
TBP_YEAST(C) IVLLIFVSG--KIVLTGA---KQREEIYQA-
TBP_HUMAN(N) TTALIFSSG--KMVCTGA---KSEEQSRLA-
TBP_HUMAN(C) IVLLIFVSG--KVVLTGA---KVRAEIYEA-
...
S4.1 S4.2 S4.3 S4.4 S4.5 L5.1 L5.2 L5.3 L5.4 L5.5 L5.6 S5.1 S5.2 S5.3 S5.4 S5.5 S5.6 S5.7 L6.1 L6.2 L6.3 L6.4 L6.5 H2.1 H2.2 H2.3 H2.4 H2.5 H2.6 H2.7 H2.8
Common TBP Numbering (CTN)
Similarly, alignments were produced for TBP-interacting factors:
TFIIA
TFIID
TFIIB (TFB)
Brf1
Brf2
Mot1
NC2
b
Bacteria
Viruses
Archaea
Eukaryotes
c
Define spatio-temporal context by selecting subsets of TBP alignment based on...
...evolutionary range
e.g.
Bacteria
Archaea
Eukaryotes
...TBP lobes
e.g.
N
C
DNA

**Supplementary Figure 2. Ancestral sequence reconstruction, common TBP-lobe numbering system and dendrogram of TBP-lobe like regions**

a) Alignment of TBP-lobe like regions in RefMSA was used to construct ancestral sequences for each of the superkingdoms i.e. bacteria, archaea and eukaryotes (see **Methods** and **Main text**). While the horizontal bars indicate TBP-lobe like sequences, blue and red colours respectively denote N-terminal and C-terminal lobes for archaea, eukaryotes and viruses. The most conserved residues in the ancestral sequences between three superkingdoms were considered as universally conserved residues (see **Methods**). The ancestral sequences were integrated with the common TBP-lobe numbering (CTN) system for further analyses. A snapshot of actual CTN assignment for a part of RefMSA is shown. In the CTN system, each residue position in RefMSA is referred as <Lobe>.<Secondary structure type and secondary structure number>.<Alignment position> (e.g. N.L5.1 refers to Phe 116 in yeast; See **Methods and above**).

b) Dendrogram of TBP-lobe like regions: TBP-lobe RefMSA was used to construct this dendrogram (see **Methods**). The dendrogram indicates that TBP-lobe like regions of bacteria and viruses form distinct clusters, suggesting their significant divergence from TBP lobes of eukaryotes and archaea. For the construction of the dendrogram, we considered only sequences from representative organisms in the RefMSA alignment. We made sure to select sequences from the RefMSA that span the whole diversity of lineages represented in the RefMSA. Poxvirus sequences were not considered for the generation of the dendrogram as they are the most divergent versions of TBP-lobe like sequences (see **Methods**).

c) Approach to uncover molecular signatures for functional innovations of TBP involved utilization of two independent contexts i.e., spatial and temporal. To identify molecular signatures of TBP, evolutionary range and TBP-lobe regions from the RefMSA were used to define the temporal and spatial contexts, respectively. Here, two insets together define a particular spatio-temporal context, wherein temporal context is confining the analyses to evolutionary range of archaea and eukaryotes. The spatial context is the C-terminal TBP-lobe centric investigations.

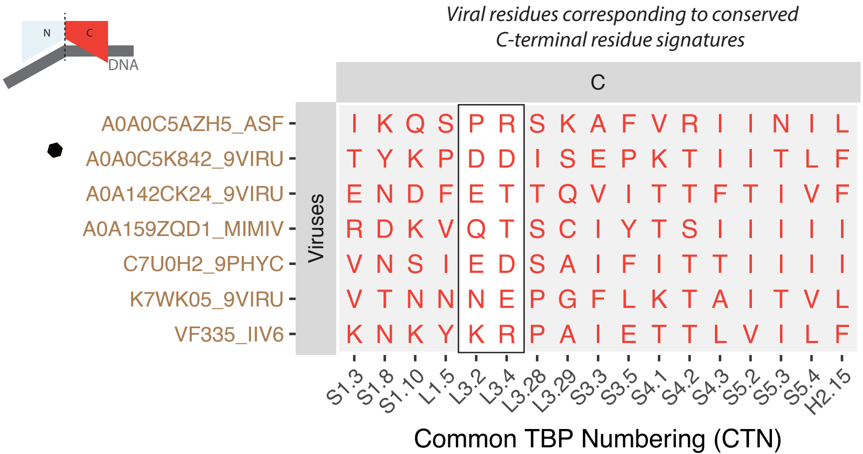
Viral residues corresponding to conserved
C-terminal residue signatures
N
C
DNA
C
Viruses
A0A0C5AZH5_ASF
A0A0C5K842_9VIRU
A0A142CK24_9VIRU
A0A159ZQD1_MIMIV
C7U0H2_9PHYC
K7WK05_9VIRU
VF335_IIV6
I K Q S P R S K A F V R I I N I L
T Y K P D D I S E P K T I I T L F
E N D F E T T Q V I T T F T I V F
R D K V Q T S C I Y T S I I I I I
V N S I E D S A I F I T T I I I I
V T N N N E P G F L K T A I T V L
K N K Y K R P A I E T T L V I L F
S1.3
S1.8
S1.10
L1.5
L3.2
L3.4
L3.28
L3.29
S3.3
S3.5
S4.1
S4.2
S4.3
S5.2
S5.3
S5.4
H2.15
Common TBP Numbering (CTN)

**Supplementary Figure 3. C-terminal lobe specific molecular signature in viral TBPs**

C-terminal lobe specific molecular signatures from archaea and eukaryotes (**Figure 3a**) mapped onto C-terminal lobes of representative viral TBPs from the RefMSA. This mapping indicates there is not high conservation of Glu or Asp at L3.2 or L3.4 in the C-terminal lobe of viral TBP-lobe sequences.

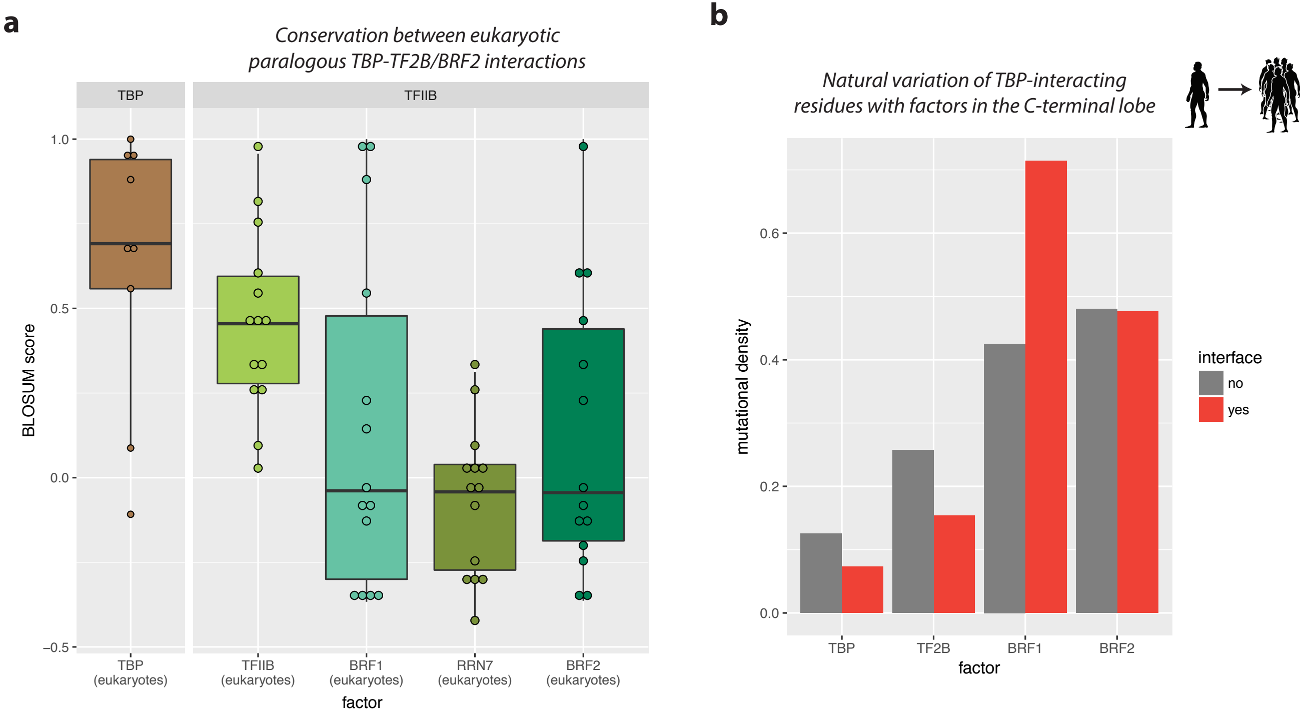
a
Conservation between eukaryotic paralogous TBP-TF2B/BRF2 interactions
TBP
TFIIB
BLOSUM score
1.0
0.5
0.0
−0.5
TBP (eukaryotes)
TFIIB (eukaryotes)
BRF1 (eukaryotes)
RRN7 (eukaryotes)
BRF2 (eukaryotes)
factor
b
Natural variation of TBP-interacting residues with factors in the C-terminal lobe
mutational density
0.6
0.4
0.2
0.0
TBP
TF2B
BRF1
BRF2
factor
interface
no
yes

**Supplementary Figure 4. Evolutionary conservation and natural variation of interaction interface residues in the C-terminal lobe of TBP**

a) The box-plots display the distribution of evolutionary conservation at the residue-level, measured in terms of normalized BLOSUM scores, of interaction mediating interface residues for eukaryotic: (i) orthologs of TBP and TFIIB and (ii) paralogs of TFIIB. The distribution of the evolutionary conservation was derived based on co-complex crystal structure (PDB: 1c9b) data of human TBP-TFIIB interactions as the reference for the comparison. The structure of human TBP-TFIIB was used to identify interaction mediating residues in both TBP and TFIIB. These residues were mapped on to equivalent residue positions in the following subsets of sequence alignments to evaluate their respective normalized BLOSUM scores across the alignment: (i) eukaryotic C-terminal TBP-lobes from RefMSA (for TBP only), (ii) TFIIB orthologs alignment in eukaryotes, (iii) alignment of TFIIIB/BRF1 orthologs, (iv) alignment of BRF2 orthologs and (v) alignment of TAF1B/Rrn7 orthologs (see **Supplementary data**). Horizontal line within each of the box plots indicates median score of conservation. TBP displays highest conservation, relative to the other interacting factors, at these interface residue positions.

b) The bar plot depicts mutation densities for natural variation of residues at the interaction interfaces of C-terminal lobe of TBP with TFIIB and its paralogs. Mutation density (rate of mutations normalized to the sequence length) bar plots has been made based on data from known co-complex structures of TBP C-terminal lobe with various factors such as TFIIB, BRF1, BRF2 (PDB: 1c9b, 6f40 and 4roc). “Red bar” indicate mutation density for the interface residues, while “gray bar” indicates the same measure for non-interface residues. The bar plots indicate that TBP (C-terminal lobe) has the least natural variation at the interface as compared to the other factors (**Methods**). Hence similar to the pattern of evolutionary conservation within eukaryotes, natural variations indicate that interface residues of TBP interacting factors does display a greater mutational tendency compared to TBP interface residues for the C-terminal lobe interactions.

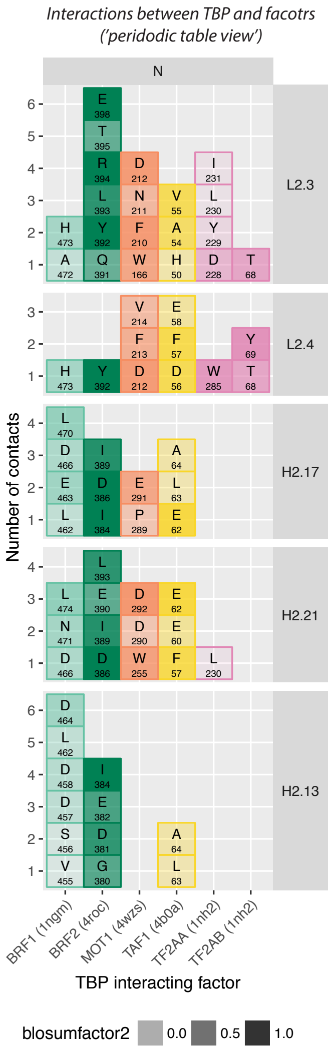

Interactions between TBP and facotrs
('peridodic table view')
N
L2.3
L2.4
H2.17
H2.21
H2.13
Number of contacts
TBP interacting factor
BRF1 (1ngm)
BRF2 (4roc)
MOT1 (4wzs)
TAF1 (4b0a)
TF2AA (1nh2)
TF2AB (1nh2)
blosumfactor2
0.0
0.5
1.0

**Supplementary Figure 5. Interaction residues of various factors that mediate interactions with the molecular signature positions in the N-terminal lobe of TBP.** The plot displays detailed view of number of interaction contacts of the 5 conserved molecular signature positions (shown in the right y-axis; see **Figure 4**) in the N-terminal lobe TBP and their interacting residues in various factors (shown in the x-axis). The darker the color of boxes higher the evolutionary conservation of that residue. These interactions are deduced based on available co-complex structures of these factors with TBP (**Figure 4** and **Methods**). The majority of the residues displayed in the plot are either acidic or aromatic residues suggesting the existence of a significant number of electrostatic interactions between N-terminal lobe of TBP and these factors (see **Figure 4**). These acidic or aromatic residues in some cases do not display a strong evolutionary conservation as these residues fall in intrinsically unstructured or disordered regions. However, while not conserving individual acidic or aromatic residues, overall they preserve negatively charged or aromatic characteristics in these residue sequence neighborhoods (see **Supplementary Figure 6** and **Figure 4**).

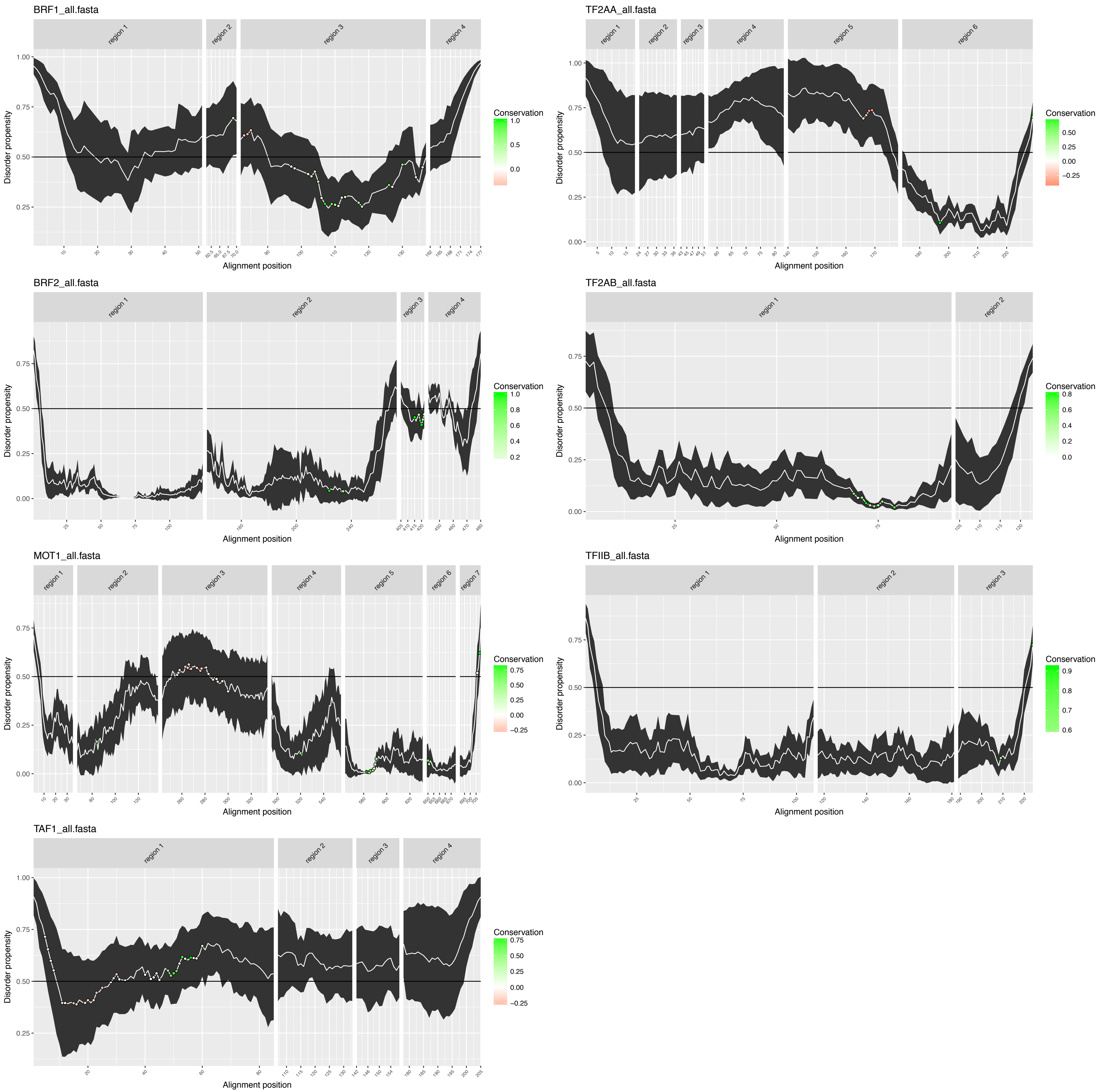
BRF1_all.fasta
region 1
region 2
region 3
region 4
Disorder propensity
Alignment position
Conservation
TF2AA_all.fasta
region 1
region 2
region 3
region 4
region 5
region 6
Disorder propensity
Alignment position
Conservation
BRF2_all.fasta
region 1
region 2
region 3
region 4
Disorder propensity
Alignment position
Conservation
TF2AB_all.fasta
region 1
region 2
Disorder propensity
Alignment position
Conservation
MOT1_all.fasta
region 1
region 2
region 3
region 4
region 5
region 6
region 7
Disorder propensity
Alignment position
Conservation
TFIIB_all.fasta
region 1
region 2
region 3
Disorder propensity
Alignment position
Conservation
TAF1_all.fasta
region 1
region 2
region 3
region 4
Disorder propensity
Alignment position
Conservation

**Supplementary Figure 6. Disorder propensities of the interacting segments of various factors that interact with the N-terminal lobe of TBP in eukaryotes.**

Each plot indicates the disorder propensity, a measure of the unstructured nature of a given residue, for interacting residues of every TBP interacting factor with N-terminal lobe of TBP. The disorder propensity is calculated using IUPRED (see **Methods**) and higher values in y-axis indicate greater tendency for being intrinsically unstructured or disordered for that given residue. Typically, values in the y-axis greater than 0.5 signify assignment of disorder tendency to a given residue. Disorder propensities across organisms, as in the alignment of TBP interacting factors (see **Supplementary data**), are shown as black shaded regions in each plot and central white lines indicate mean value of disorder propensity for the respective positions (across organisms). The residue conservation is indicated by BLOSUM score is shown as green for high conservation to red for poor conservation.

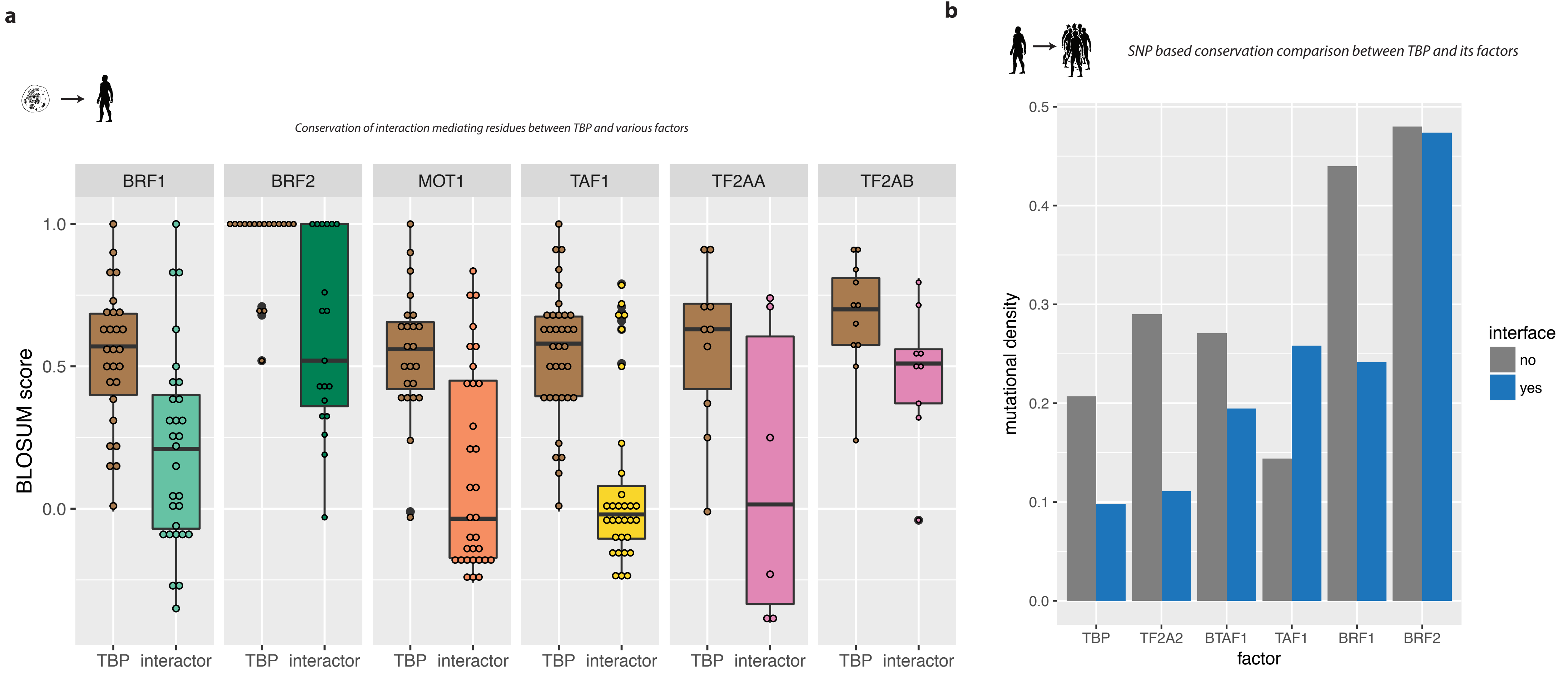
a
Conservation of interaction mediating residues between TBP and various factors
BRF1
BRF2
MOT1
TAF1
TF2AA
TF2AB
BLOSUM score
1.0
0.5
0.0
TBP
interactor
b
SNP based conservation comparison between TBP and its factors
mutational density
0.5
0.4
0.3
0.2
0.1
0.0
TBP
TF2A2
BTAF1
TAF1
BRF1
BRF2
factor
interface
no
yes

**Supplementary Figure 7. Evolutionary conservation and natural variation of interaction interface residues at TBP N-terminal lobe**

a) Evolutionary conservation, measured in terms of BLOSUM score, of interaction mediating interfaces residues for TBP and its various interacting factors at the N-terminal lobe of TBP. This is identified based on co-complex structure data of TBP with various factors; in particular, the extent of evolutionary conservation or divergence for various intermediating residues in various factors are evaluated based on sequence alignment (see **Supplementary data**). It should be noted that conservation plots are made for TBP and its interacting factors with an identical phylogenetic range in order to have a meaningful comparison. Horizontal lines within the box plot indicate median score of conservation for each factor's interface residues. In the majority of cases, TBP displays better evolutionary conservation than its interaction partners at the interaction mediating interface residue positions.

b) The bar plots of natural variation of residues measured as mutational densities (see **Methods** and **above**) at the interface for various factors that interact with the N-terminal lobe of TBP. "Blue bars" indicate mutation density for the residue at the interface mediating interactions, while "gray bar" represents mutation density for non-interface residues. The barplots indicate TBP has the least natural variation at the interface as compared to other factors. Hence, similar to the pattern of evolutionary conservation and natural variation of interaction interfaces at the C-terminal lobe of TBP, these plots indicate that the interface residues of TBP interacting factors does display a greater mutational frequency compared to TBP interface residues.

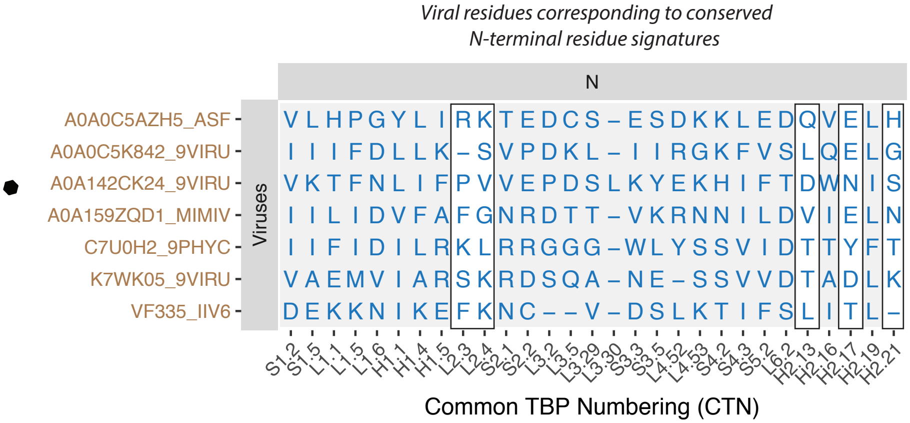

Viral residues corresponding to conserved
N-terminal residue signatures
N
Viruses
A0A0C5AZH5_ASF
A0A0C5K842_9VIRU
A0A142CK24_9VIRU
A0A159ZQD1_MIMIV
C7U0H2_9PHYC
K7WK05_9VIRU
VF335_IIV6
V L H P G Y L I R K T E D C S – E S D K K L E D Q V E L H
I I I F D L L K – S V P D K L – I I R G K F V S L Q E L G
V K T F N L I F P V V E P D S L K Y E K H I F T D W N I S
I I L I D V F A F G N R D T T – V K R N N I L D V I E L N
I I F I D I L R K L R R G G G – W L Y S S V I D T T Y F T
V A E M V I A R S K R D S Q A – N E – S S V V D T A D L K
D E K K N I K E F K N C – – V – D S L K T I F S L I T L –
S1.2 S1.5 L1.1 L1.5 L1.6 H1.1 H1.4 H1.5 L2.3 L2.4 S2.1 S2.2 L3.2 L3.5 L3.29 L3.30 S3.3 S3.5 L4.52 L4.53 S4.2 S4.3 S5.2 L6.2 H2.13 H2.16 H2.17 H2.19 H2.21
Common TBP Numbering (CTN)

**Supplementary Figure 8. Molecular signature residue positions mapped to the N-terminal lobe of TBP of dsDNA viruses.**

Highly conserved residue positions in the N-terminal lobe of TBP (see **Figure 4**) mapped on their dsDNA viruses orthologs. The five residue positions that are either positively charged positions or contains asparagine are indicated within rectangular boxes. It is clear from these indicated positions that, in the viruses, these positions, by and large, are devoid of positively charged residues. Given the positively charged residues are critical for mediating TBP interactions (**Figure 4** and **Supplementary Figure 5**) with various factors, which in turn function as regulatory controls, this is suggestive that viruses potentially avoid the host regulatory controls. These regulatory controls could otherwise place restrictions on functions of viral TBPs in their respective hosts.

**a**

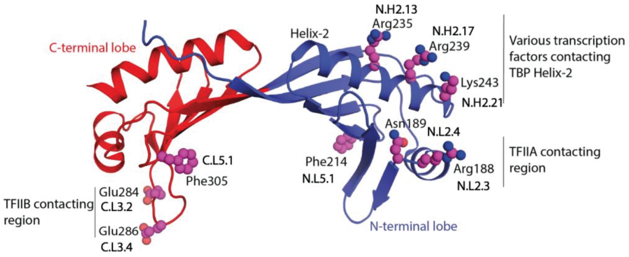


**b**

| Mutation | Organism | Phenotype | Lobe | CTN | Signature Type | Pubmed ID |
|---|---|---|---|---|---|---|
| F116Y | Yeast | Electrophoretic mobility shift assay with promoter DNA probe | N | N.L5.1 | Universal | 2015629 |
| F116A | | | | | | 24865972 |
| F207A | | | C | C.L5.1 | | 24865972 |
| F214A | Human | | N | N.L5.1 | | 16179647 |
| F305A and F305K | | | C | C.L5.1 | | 16179647 |
| F288K and F288A | Human | Significant reduction in transcription activity (*in vitro*) at one or more promoters | C | C.L3.27 | | 12535529 |
| F305A and F305K | | | C | C.L5.1 | | 12535529 |
| F305A | Human | Significant reduction in basal transcription (*in vitro*) | C | C.L5.1 | | 8843200 |
| S307F | | | C | C.L5.3 | | 8843200 |
| K316E | | | C | C.L6.1 | | 8843200 |
| L165E | | | N | N.S1.2 | N-lobe specific | 8843200 |
| D179R | | | N | N.L1.6 | | 8843200 |
| A184E | | | N | N.H1.5 | | 8843200 |
| R188E | | | N | N.L2.3 | | 8843200 |
| R188E | Human | Significant partial reduction in transcription activity (*in vitro*) at one or more promoters | N | N.L2.3 | | 12535529 |
| N189E | | | N | N.L2.4 | | 12535529 |
| N189E | Human | Significant reduction in basal transcription (*in vitro*) | N | N.L2.4 | | 8843200 |
| A190E | | | N | N.S2.1 | | 8843200 |
| E191K | Human | Significant reduction in transcription activity (*in vitro*) at one or more promoters | N | N.S2.2 | | 12535529 |
| E191R | Human | Partial reduction in basal transcription (*in vitro*) | N | N.S2.2 | | 8843200 |
| E93R | Yeast | Gene expression changes in yeast system and perturbation to interaction with TFIIA | N | N.S2.2 | | 17407552 |
| N193R | Human | Significant reduction in basal transcription (*in vitro*) | N | N.L3.2 | | 8843200 |
| E206R | Human | Marginal reduction in basal transcription (*in vitro*) | N | N.L4.52 | | 8843200 |
| R235E | Human | Potential increase in basal transcription (*in vitro*) | N | N.H2.13 | | 8843200 |
| R239S | | | N | N.H2.17 | | 8843200 |
| K145E | Yeast | Gene expression changes in yeast system and perturbation to interaction with Mot1p | N | N.H2.21 | | 17407552 |
| S261E | Human | Significant reduction in basal transcription (*in vitro*) | C | C.S1.8 | C-lobe specific | 8843200 |
| D263R | | | C | C.S1.10 | | 8843200 |
| E284R | | | C | C.L3.2 | | 8843200 |
| E284A and E284R | Human | Significant reduction in transcription activity (*in vitro*) at one or more promoters | C | C.L3.2 | | 12535529 |
| E286R | | | C | C.L3.4 | | 12535529 |
| E286R | Human | Significant reduction in basal transcription (*in vitro*) | C | C.L3.4 | | 8843200 |

**Supplementary Figure 9. Mapping of Mutational data.**

a) Molecular signature residues of TBP and experimental data. Highlighted residues that are prominent molecular signatures for interactions that have support from experimental data. For more comprehensive data please see the table in b) below.

b) Mutational data that provide support for molecular signatures.

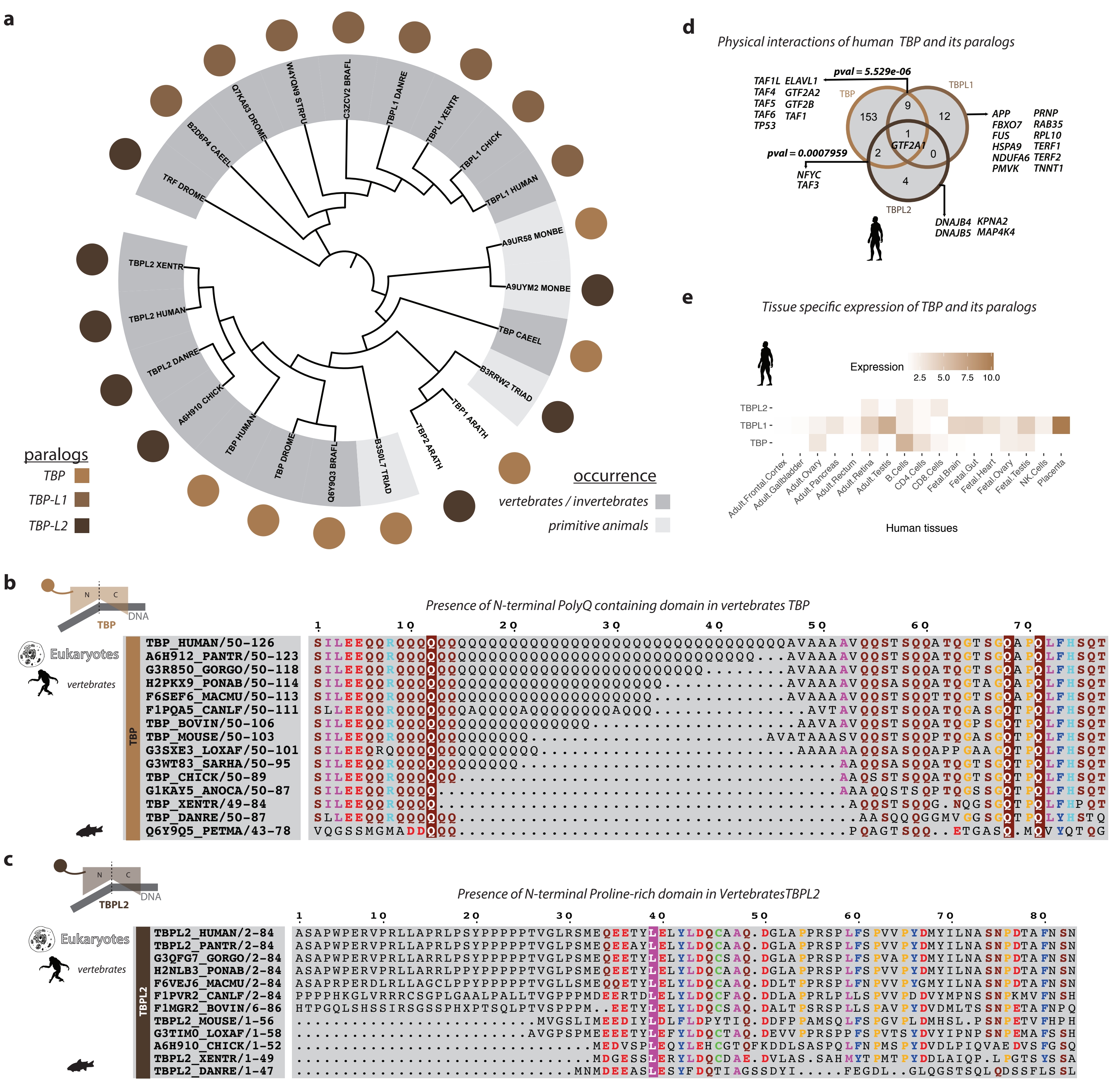
a
paralogs
TBP
TBP-L1
TBP-L2
occurrence
vertebrates / invertebrates
primitive animals
TRF DROME
B2D6P4 CAEEL
Q7KA83 DROME
W4YQN9 STRPU
C3ZCV2 BRAFL
TBPL1 DANRE
TBPL1 XENTR
TBPL1 CHICK
TBPL1 HUMAN
A9UR58 MONBE
A9UYM2 MONBE
TBP CAEEL
B3RRW2 TRIAD
TBP1 ARATH
TBP2 ARATH
B3S0L7 TRIAD
Q6Y9Q3 BRAFL
TBP DROME
TBP HUMAN
A6H910 CHICK
TBPL2 DANRE
TBPL2 HUMAN
TBPL2 XENTR
d
Physical interactions of human TBP and its paralogs
pval = 5.529e-06
TAF1L ELAVL1
TAF4 GTF2A2
TAF5 GTF2B
TAF6 TAF1
TP53
TBP
TBPL1
TBPL2
153
9
12
1
GTF2A1
2
0
4
pval = 0.0007959
NFYC
TAF3
APP PRNP
FBXO7 RAB35
FUS RPL10
HSPA9 TERF1
NDUFA6 TERF2
PMVK TNNT1
DNAJB4 KPNA2
DNAJB5 MAP4K4
e
Tissue specific expression of TBP and its paralogs
Expression
2.5 5.0 7.5 10.0
TBPL2
TBPL1
TBP
Adult.Frontal.Cortex
Adult.Gallbladder
Adult.Ovary
Adult.Pancreas
Adult.Rectum
Adult.Retina
Adult.Testis
B.Cells
CD4.Cells
CD8.Cells
Fetal.Brain
Fetal.Gut
Fetal.Heart
Fetal.Ovary
Fetal.Testis
NK.Cells
Placenta
Human tissues
b
N
C
TBP
DNA
Presence of N-terminal PolyQ containing domain in vertebrates TBP
Eukaryotes
vertebrates
TBP
TBP_HUMAN/50-126
A6H912_PANTR/50-123
G3R850_GORGO/50-118
H2PKX9_PONAB/50-114
F6SEF6_MACMU/50-113
F1PQA5_CANLF/50-111
TBP_BOVIN/50-106
TBP_MOUSE/50-103
G3SXE3_LOXAF/50-101
G3WT83_SARHA/50-95
TBP_CHICK/50-89
G1KAY5_ANOCA/50-87
TBP_XENTR/49-84
TBP_DANRE/50-87
Q6Y9Q5_PETMA/43-78
c
N
C
TBPL2
DNA
Presence of N-terminal Proline-rich domain in VertebratesTBPL2
Eukaryotes
vertebrates
TBPL2
TBPL2_HUMAN/2-84
TBPL2_PANTR/2-84
G3QFG7_GORGO/2-84
H2NLB3_PONAB/2-84
F6VEJ6_MACMU/2-84
F1PVR2_CANLF/2-84
F1MGR2_BOVIN/6-86
TBPL2_MOUSE/1-56
G3TIM0_LOXAF/1-58
A6H910_CHICK/1-52
TBPL2_XENTR/1-49
TBPL2_DANRE/1-47

**Supplementary Figure 10. Conserved regions, protein-protein interactions and tissue expression in TBP and its paralogs.**

a) Dendrograms depicting relationship between TBP and its paralogs (**Supplementary Data**). These dendrograms have been constructed using representative sequences that capture diversity of animal lineages in which these proteins were detected (see **Methods**). TBP and TBPL2 are found in early or primitive animals, while TBPL1 is found only in invertebrates and vertebrates suggesting that TBPL1 is the most recent paralog of TBP as well as the most divergent.
b) Multiple sequence alignment of N-terminal PolyGln stretches containing regions of TBP. Only a part of the alignment of this evolutionary conserved region is shown (see **Supplementary Data** for full alignment of this domain). This region is present only in vertebrates. It is clear that there is an approximate linear increase in sequence length of PolGln (PolyQ) stretches in TBP from zebra fish to humans.
c) Multiple sequence alignment of N-terminal proline rich regions of TBPL2. Only a part of the alignment of this evolutionary conserved region is shown (see **Supplementary data** for full alignment of this region). This region is present only in vertebrates and particularly prominent in mammals.
d) Physical interactions of human TBP, TBPL1 and TBPL2. While the data is sparse for TBPL1 and TBPL2, their shared interactions with TBP appears statistically significant. However, albeit with limited protein-protein interaction data, we observe the existence of unique interaction partners of TBPL1 and TBPL2. This is suggestive of potential distinct function contexts for TBPL1 and TBPL2. Given the higher sequence divergence and distinct pattern of protein expression across human tissues of TBPL1, interaction divergence could be additional contributing factor for functional divergence between TBP and TBPL1 (see **Supplementary Figure 10e**). The physical interaction data has been obtained from BIOGRID and Intact databases (Oughtred et al, 2019; Kerrien et al, 2012).
e) Protein level expression of human TBP, TBPL1 and TBPL2 across 17 different tissues (Kim et al, 2014). TBPL1 is almost ubiquitously expressed in majority of these tissues, while TBP and TBPL2 are more restricted. This may be due to their low-level of expression, which might be hard to detect by high throughput methods. Nevertheless, TBPL1 appears to have a divergent expression pattern indicated by Pearson correlation of 0.2 with TBP. This along with the earlier observation that TBPL1 is most divergent of the paralogs of TBP is suggestive of distinct functional niche of TBPL1. TBPL2 has a robust co-expression with TBP with a Pearson correlation of 0.7 and this might suggest some level of functional overlap between TBP and TBPL2 for e.g. DNA sequence recognition, protein-protein interaction, etc.

## Analysis Overview

| | |
|---|---|
| Comparison on N- and C-terminal lobes of TBP | ✓ |
| Evolution of interactions | ✓ |
| Region of main focus in TBP | All regions in TBP were considered |
| Number of adaptors studied | 8 adaptors |
| Number of sequences analyzed | ~400 sequence domains |
| Evolutionary range covered | Bacteria, Archaea, Eukaryotes and Viruses were considered |
| Reduction of potential bias | Representatives from every lineage were carefully considered to minimise bias in discovery of signature residues |
| Number of structures analyzed | 10 co-complex structures |
| Number of molecular signature residues identified | 5 residues for ds nucleic acid binding<br>2 residues for C-lobe interactions<br>5 residues for N-lobe interactions |
| Integration of additional datasets | Genome-scale data such as transcriptomic and proteomics data were integrated |
| Support from mutational data (new analysis) | ✓ |
| Distinguishing orthologs and paralogs | ✓ |
| Common numbering system | ✓ |
| Comprehensive resource of alignments of TBP, TBP-like proteins and its interaction partners | ✓ |
| Approach applicable to other protein families | ✓ |

**Supplementary Figure 11: Overview of datasets used and analyses performed in Ravarani *et al.***

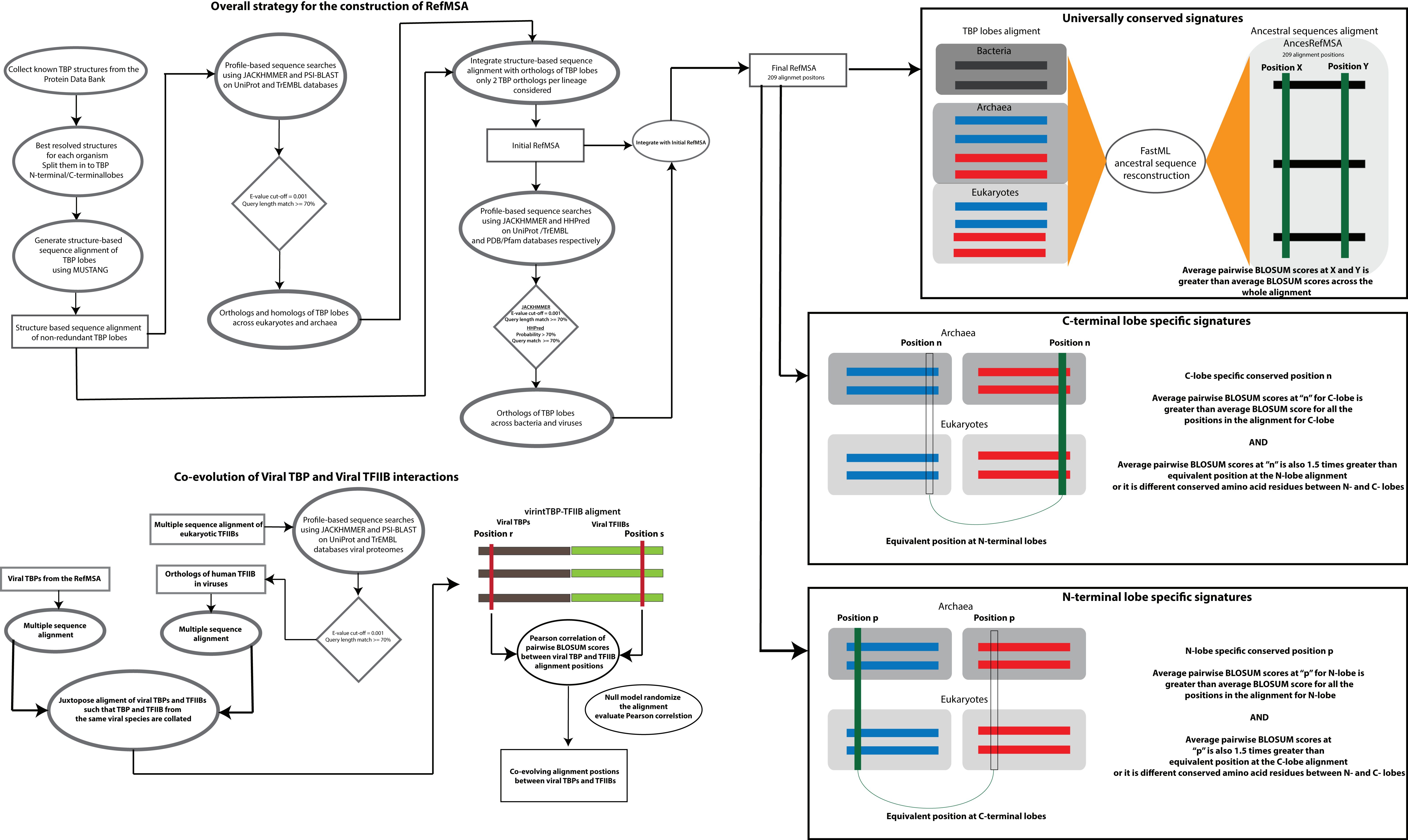
Overall strategy for the construction of RefMSA
Collect known TBP structures from the Protein Data Bank
Best resolved structures for each organism Split them in to TBP N-terminal/C-terminallobes
Generate structure-based sequence alignment of TBP lobes using MUSTANG
Structure based sequence alignment of non-redundant TBP lobes
Profile-based sequence searches using JACKHMMER and PSI-BLAST on UniProt and TrEMBL databases
E-value cut-off = 0.001 Query length match >= 70%
Orthologs and homologs of TBP lobes across eukaryotes and archaea
Integrate structure-based sequence alignment with orthologs of TBP lobes only 2 TBP orthologs per lineage considered
Initial RefMSA
Integrate with Initial RefMSA
Profile-based sequence searches using JACKHMMER and HHPred on UniProt /TrEMBL and PDB/Pfam databases respectively
JACKHMMER E-value cut-off = 0.001 Query length match >= 70% HHPred Probability > 70% Query match >= 70%
Orthologs of TBP lobes across bacteria and viruses
Final RefMSA 209 alignmet positons
Universally conserved signatures
TBP lobes aligment
Bacteria
Archaea
Eukaryotes
FastML ancestral sequence resconstruction
Ancestral sequences aligment AncesRefMSA
209 alignment positions
Position X
Position Y
Average pairwise BLOSUM scores at X and Y is greater than average BLOSUM scores across the whole alignment
C-terminal lobe specific signatures
Archaea
Position n
Position n
Eukaryotes
Equivalent position at N-terminal lobes
C-lobe specific conserved position n
Average pairwise BLOSUM scores at "n" for C-lobe is greater than average BLOSUM score for all the positions in the alignment for C-lobe
AND
Average pairwise BLOSUM scores at "n" is also 1.5 times greater than equivalent position at the N-lobe alignment or it is different conserved amino acid residues between N- and C- lobes
N-terminal lobe specific signatures
Archaea
Position p
Position p
Eukaryotes
Equivalent position at C-terminal lobes
N-lobe specific conserved position p
Average pairwise BLOSUM scores at "p" for N-lobe is greater than average BLOSUM score for all the positions in the alignment for N-lobe
AND
Average pairwise BLOSUM scores at "p" is also 1.5 times greater than equivalent position at the C-lobe alignment or it is different conserved amino acid residues between N- and C- lobes
Co-evolution of Viral TBP and Viral TFIIB interactions
Multiple sequence alignment of eukaryotic TFIIBs
Profile-based sequence searches using JACKHMMER and PSI-BLAST on UniProt and TrEMBL databases viral proteomes
E-value cut-off = 0.001 Query length match >= 70%
Viral TBPs from the RefMSA
Orthologs of human TFIIB in viruses
Multiple sequence alignment
Multiple sequence alignment
Juxtapose aligment of viral TBPs and TFIIBs such that TBP and TFIIB from the same viral species are collated
virintTBP-TFIIB aligment
Viral TBPs
Viral TFIIBs
Position r
Position s
Pearson correlation of pairwise BLOSUM scores between viral TBP and TFIIB alignment positions
Null model randomize the alignment evaluate Pearson correlstion
Co-evolving alignment postions between viral TBPs and TFIIBs

**Supplementary Figure 12: Overview of Methods.**
