# Supplementary material for: Molecular determinants underlying functional innovations of TBP and their impact on transcription initiation
Source: Nat Commun. 2020 May 13;11:2384. doi: 10.1038/s41467-020-16182-z (PMC7221094; doi:10.1038/s41467-020-16182-z)
Supplement: Supplementary file 3 — Description of Additional Supplementary Files [file 41467_2020_16182_MOESM3_ESM.docx]

**Description of Additional Supplementary Files**

File name: Supplementary Data 1
Description: TBP Multiple Sequence Alignment

File name: Supplementary Data 2
Description: Viral TBP Multiple Sequence Alignment

File name: Supplementary Data 3
Description: TFIIB and its Homologs Multiple Sequence Alignment

File name: Supplementary Data 4
Description: Multiple sequence alignment of TFIIB and Sigma factor homologous regions

File name: Supplementary Data 5
Description: TBP and TFIIB in Viral Genomes

File name: Supplementary Data 6
Description: Viral TBP and TFIIB Co-evolution

File name: Supplementary Data 7
Description: TBP Interacting Factors alignment within Eukaryotes  (BRF1, BRF2, TFIIB, TFIIA, MOT1/BTAF1, NC2, TAF1/TFIID)

File name: Supplementary Data 8
Description: Overlap in TBP residues between various TBP Interacting Factors

File name: Supplementary Data 9
Description: Multiple Sequence Alignment of TBP, TBPL1 and TBPL2 orthologs in animals

File name: Supplementary Data 10
Description: Dendrogram of TBPLs at the level of TBP-lobe regions

File name: Supplementary Data 11
Description: Multiple sequence alignment of whole PolyGln stretches containing region of TBP

File name: Supplementary Data 12
Description: Multiple sequence alignment of whole Proline-rich stretches containing region of TBPL2

File name: Supplementary Data 13
Description: Non-Missense Mutations in PolyQ containing region in TBP

File name: Supplementary Data 14
Description: Multiple Sequence Alignment of MED8

File name: Supplementary Data 15
Description: Multiple Sequence Alignment of MYC

File name: Supplementary Data 16
Description: Overview of Methods
