# Supplementary figures and images for: Molecular determinants underlying functional innovations of TBP and their impact on transcription initiation
Source: Nat Commun. 2020 May 13;11:2384. doi: 10.1038/s41467-020-16182-z (PMC7221094; doi:10.1038/s41467-020-16182-z)

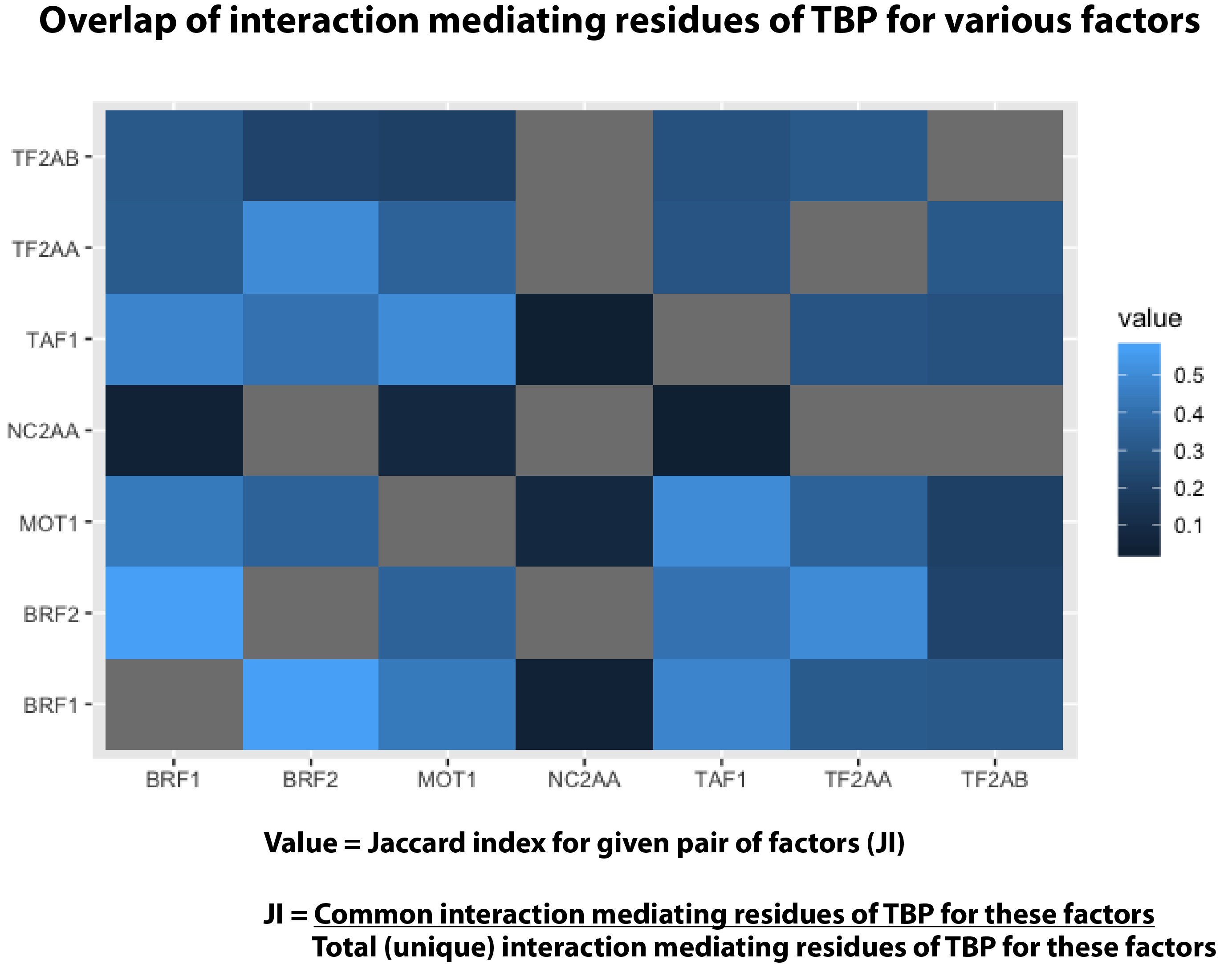

Supplement: Supplementary file 4 — Supplementary Data 1-16 [file 41467_2020_16182_MOESM4_ESM.zip › 5_Ravarani_et_al_TBP_functional_innovation_Suppl_Data/8. Overlap in TBP residues between various TBP Interacting Factors/Overlap_of_factors.png]

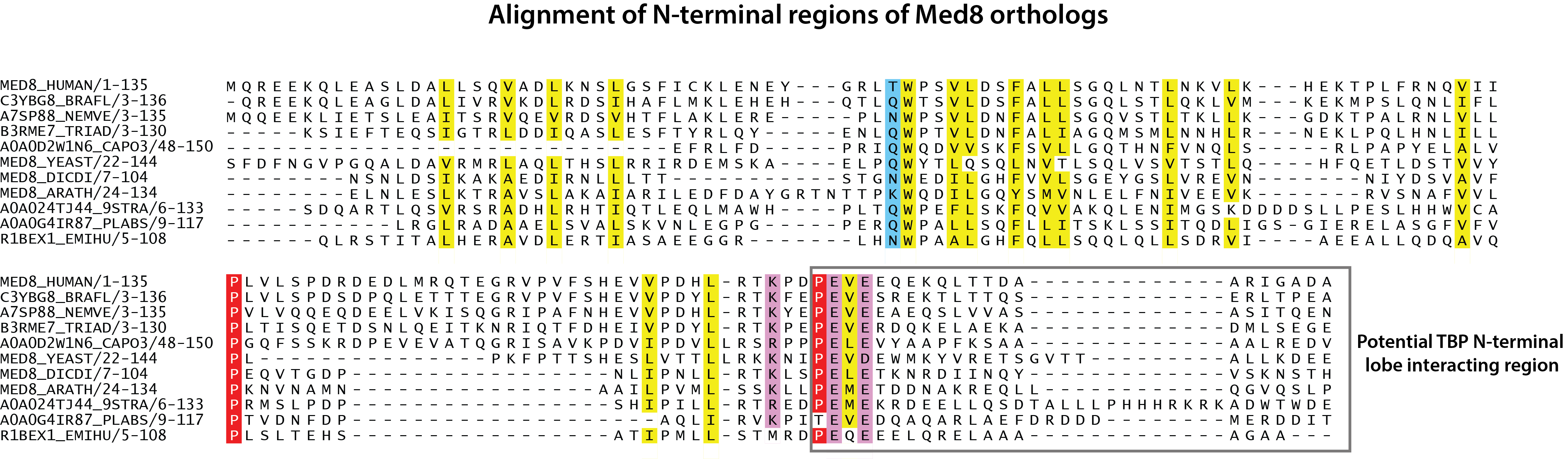

Supplement: Supplementary file 4 — Supplementary Data 1-16 [file 41467_2020_16182_MOESM4_ESM.zip › 5_Ravarani_et_al_TBP_functional_innovation_Suppl_Data/14. Multiple Sequence Alignment of MED8/med8_nterm_alignment.bound.png]

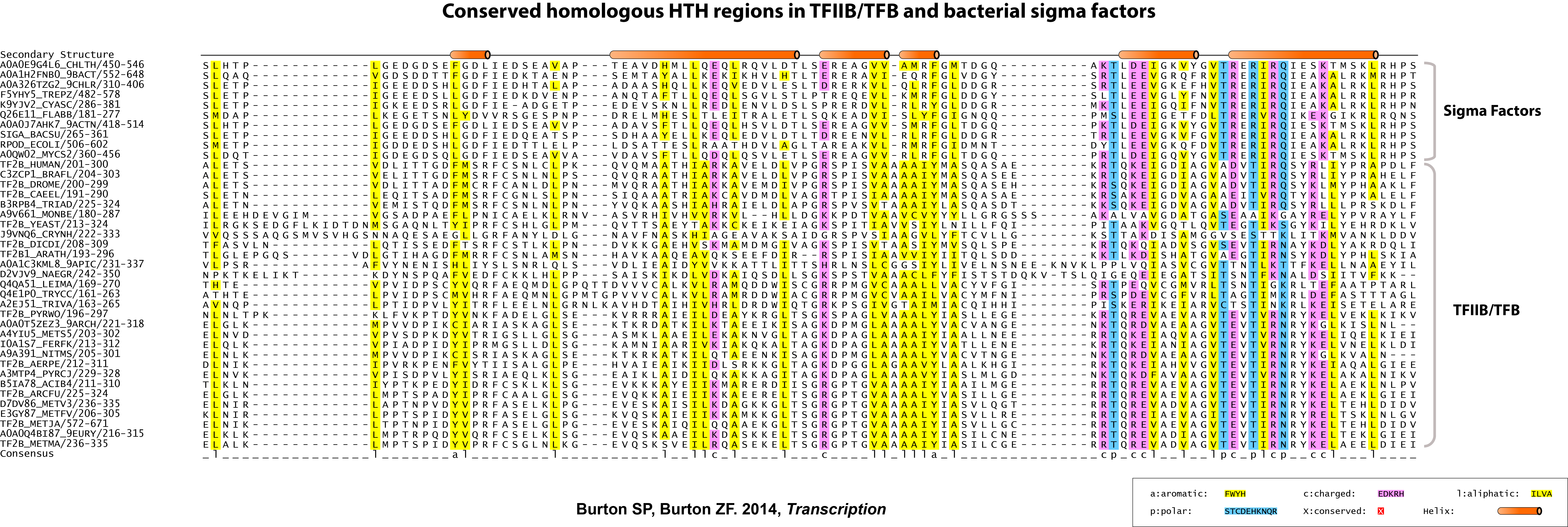

Supplement: Supplementary file 4 — Supplementary Data 1-16 [file 41467_2020_16182_MOESM4_ESM.zip › 5_Ravarani_et_al_TBP_functional_innovation_Suppl_Data/4. Multiple sequence alignment of TFIIB and Sigma factor homologous regions/TF2B_Sigmafactor_homologous_regions_final_suppl.png]

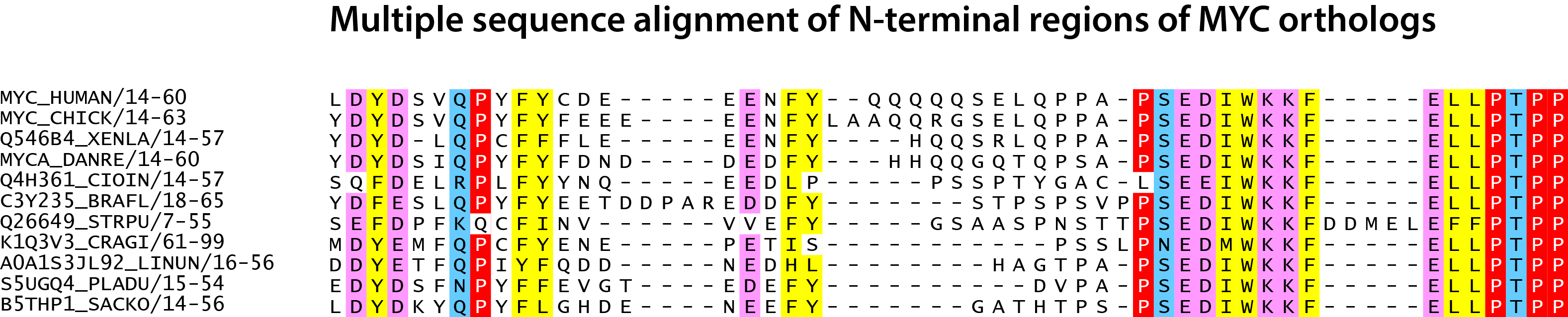

Supplement: Supplementary file 4 — Supplementary Data 1-16 [file 41467_2020_16182_MOESM4_ESM.zip › 5_Ravarani_et_al_TBP_functional_innovation_Suppl_Data/15. Multiple Sequence Alignment of MYC/MYC_align.png]

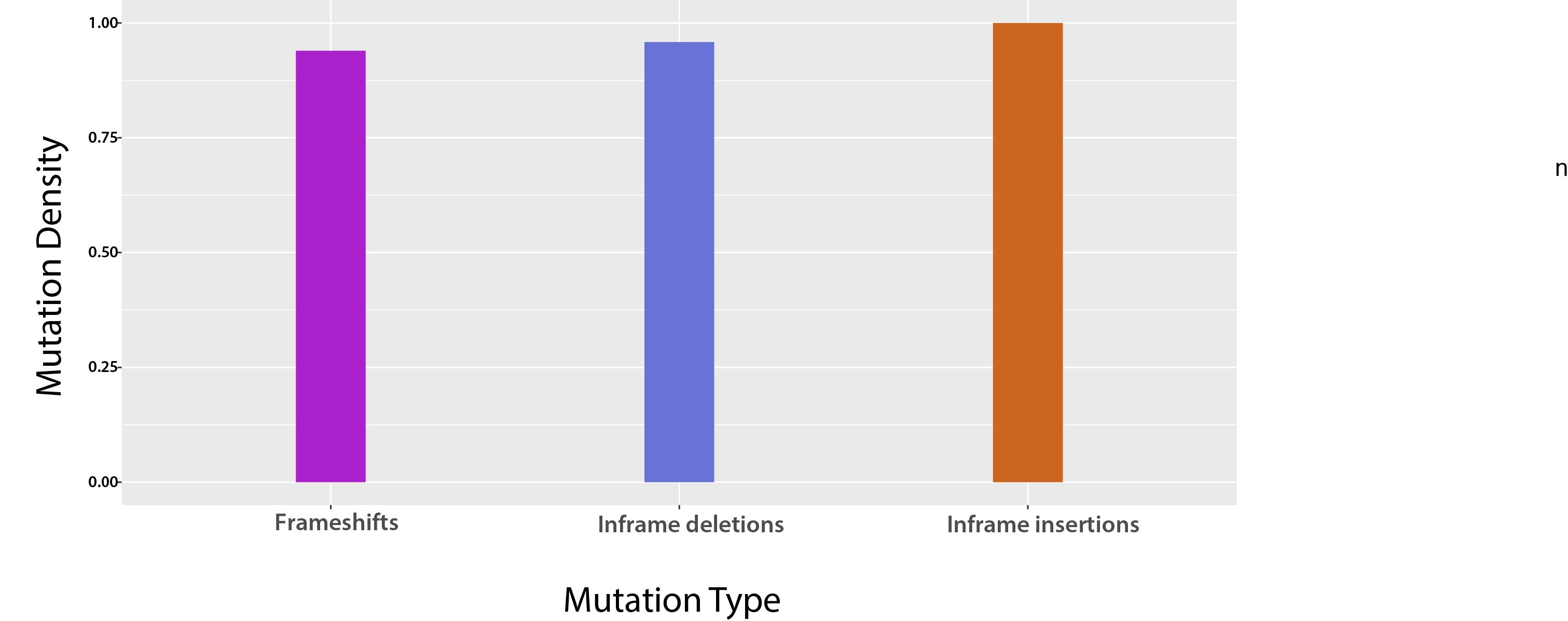

Supplement: Supplementary file 4 — Supplementary Data 1-16 [file 41467_2020_16182_MOESM4_ESM.zip › 5_Ravarani_et_al_TBP_functional_innovation_Suppl_Data/13. Non-Missense Mutations in PolyQ containing region in TBP/PolyQdomain_TBP_nonmisense_mutations.jpg]
